# An Extremely Sensitive Ultra-High Throughput Growth Selection Assay for the Identification of Amidase Activity

**DOI:** 10.1007/s00253-024-13233-z

**Published:** 2024-06-24

**Authors:** Yannick Branson, Bjarne Schnell, Celine Zurr, Thomas Bayer, Christoffel P.S. Badenhorst, Ren Wei, Uwe T. Bornscheuer

**Affiliations:** Department of Biotechnology & Enzyme Catalysis, Institute of Biochemistry, University of Greifswald, 17487 Greifswald, Germany

**Keywords:** Amidases, *p*-aminobenzoic acid, Growth selection, Hydrolases, Ultra-high throughput screening

## Abstract

**Abstract:**

In the last decades, biocatalysis has offered new perspectives for the synthesis of (chiral) amines, which are essential building blocks for pharmaceuticals, fine and bulk chemicals. In this regard, amidases have been employed due to their broad substrate scope and their independence from expensive cofactors. To expand the repertoire of amidases, tools for their rapid identification and characterization are greatly demanded. In this work an ultra-high throughput growth selection assay based on the production of the folate precursor *p*-aminobenzoic acid (PABA) is introduced to identify amidase activity. PABA-derived amides structurally mimic the broad class of commonly used chromogenic substrates derived from *p*-nitroaniline. This suggests that the assay should be broadly applicable for the identification of amidases. Unlike conventional growth selection assays that rely on substrates as nitrogen or carbon source, our approach requires PABA in sub-nanomolar concentrations, making it exceptionally sensitive and ideal for engineering campaigns that aim at enhancing amidase activities from minimally active starting points, for example. The presented assay offers flexibility in the adjustment of sensitivity to suit project-specific needs using different expression systems and fine-tuning with the antimetabolite sulfathiazole. Application of this PABA-based assay facilitates the screening of millions of enzyme variants on a single agar plate within two days, without the need for laborious sample preparation or expensive instruments, with transformation efficiency being the only limiting factor.

**Key points:**

*• Ultra-high throughput assay (tens of millions on one agar plate) for amidase screening*

*• High sensitivity by coupling selection to folate instead of carbon or nitrogen source*

*• Highly adjustable in terms of sensitivity and expression of the engineering target*

**Supplementary Information:**

The online version contains supplementary material available at 10.1007/s00253-024-13233-z.

## Introduction

Chiral amines are key building blocks for a vast number of pharmaceuticals and agrochemicals with considerable market size. Thus, their biocatalytic synthesis has been the focus of both academia and the pharmaceutical and chemical industries (Höhne and Bornscheuer 2009; Kroutil et al. 2013; Reetz 2013; Ghislieri and Turner 2014). Transaminases (Koszelewski et al. 2010; Steffen-Munsberg et al. 2015; Slabu et al. 2017), amine dehydrogenases (Ducrot et al. 2021), imine reductases (Mangas-Sanchez et al. 2017), monoamine oxidases (Batista et al. 2018), and ammonia lyases (Parmeggiani et al. 2018) play crucial roles in enzymatic amine synthesis and transformation. Lubberink et al. provide a comprehensive summary of these studies (Lubberink et al. 2023). The hydration of acrylonitrile to acrylamide developed by Nitto Chemical Industry (Osaka, Japan) through the application of a nitrile hydratase, or the synthesis of various enantiomerically pure amines through lipase-catalyzed acylation with ethyl methoxyacetate by BASF AG (Ludwigshafen, Germany), are some prominent examples of large-scale processes in the biocatalytic production of amides and amines, respectively (Yamada and Kobayashi 1996; Balkenhohl et al. 1997). Hydrolases have been of particular interest due to their broad substrate scope, high stereoselectivity, and their easy application since they do not depend on (expensive) cofactors (Bornscheuer and Kazlauskas 2005). Among hydrolases, amidases have been used for the synthesis of α-substituted acids, α-amino acids, and chiral amides (Lin et al. 2019; Wu et al. 2020) yielding pharmaceuticals like marimastat, idrapril, acetohydroxamic acid, and fatty hydroxamic acids (Hamer et al. 1996; Muri et al. 2002; Fragoso et al. 2012). Amidases have also been even applied as biosensors for toxic amides from industrial effluents in wastewater treatment (Silva et al. 2009). However, to meet industrial needs regarding regio- and stereoselectivity, substrate scope, operational stability, or substrate/product inhibition, target enzymes regularly undergo extensive protein engineering through rational design or directed evolution, as for both Sitagliptin and Boceprevir manufacture by Merck & Co. Inc. and Codexis (Mijts et al. 2010; Savile et al. 2010; Desai 2011; Li et al. 2012; Wu et al. 2021). The first involves site-directed mutagenesis and requires sufficient structural information, ideally including the catalytic mechanism (Bornscheuer and Pohl 2001; Bornscheuer et al. 2012; Reetz 2013). Advances in bioinformatics assist the identification of mutational hot spots and interactions between amino acids not necessarily close to each other and away from the active site (Buller et al. 2023). Based on these predictions, only a small number of targeted point mutations or site-saturation mutagenesis reactions (semi-rational design) are necessary to improve performance. Complementary, directed evolution requires almost no information about an enzyme and follows the principle of iterative cycles of random mutagenesis and screening. Libraries of up to > 10^10^ variants are generated by error-prone PCR or DNA shuffling (Spee et al. 1993; Stemmer 1994b; Stemmer 1994a), for example, with library size essentially limited only by transformation efficiency (Bornscheuer et al. 2019). Therefore, a major challenge of directed evolution lies in the screening of massive libraries within a reasonable amount of time and effort (Bornscheuer 1998; Bornscheuer and Pohl 2001; Bornscheuer et al. 2012; Arnold 2019). Many recent developments in high-throughput screening methods have been achieved recently and are thoroughly compared in several reviews (Xiao et al. 2015; Packer and Liu 2015; Bunzel et al. 2018; Morrison et al. 2020; Buller et al. 2023). Growth selection assays are a class of ultra-high throughput screenings (Fibinger et al. 2015; Wu et al. 2022), where enzyme performance is coupled to cell metabolism so that only cells with a desired activity can survive and outgrow other variants, resulting in an impressive throughput with relatively low effort which basically is limited only by transformation, resulting in a throughput of up to 10^10^ in *Escherichia coli* (*E. coli)*. Only *in vitro* approaches, reaching throughputs of up to 10^14^, surpass the performance of growth selection significantly, however, accompanied by the disadvantage of limited applications on screening for catalysts, because they usually depend on molecular binding events (Newton et al. 2020). Recently, Wu et al. published a growth selection assay successfully applied to search for amine-converting or forming enzymes (Wu et al. 2022). In this and many other cases, the growth of the strain exclusively relies on producing substantial amounts of a carbon or nitrogen source. Therefore, this kind of growth selection can be considered to have low sensitivity, making it ideal for finding highly active enzymes. When starting from an enzyme with no or very low activity, growth selections may therefore be too stringent for identification of more active variants in the first rounds of directed evolution. In cases where a novel activity must be created from an inactive scaffold, the combination of ultra-high throughput and extreme sensitivity would be of great advantage, since initial variants with slightly improved activity could then be identified. This is the case in projects exploring the promiscuous activity of an enzyme, like the identification of amidase activity of promiscuous esterases for pharmaceutical or chemical industrial applications (Kourist et al. 2008; Jung et al. 2013; Hackenschmidt et al. 2014; Kürten et al. 2016; Galmés et al. 2020). A similar application is the engineering of polyester hydrolases (polyesterases) to degrade related polymers like polyamides (Biundo et al. 2019). If the promiscuous (poly)-amidase activity of polyesterases, which already have promiscuous polymer-binding properties (e.g., cutinases can degrade PET), could be significantly increased, efficient polyamidases could perhaps be created (Chen et al. 2013; Sulaiman et al. 2014; Biundo et al. 2019).

**Fig. 1:**
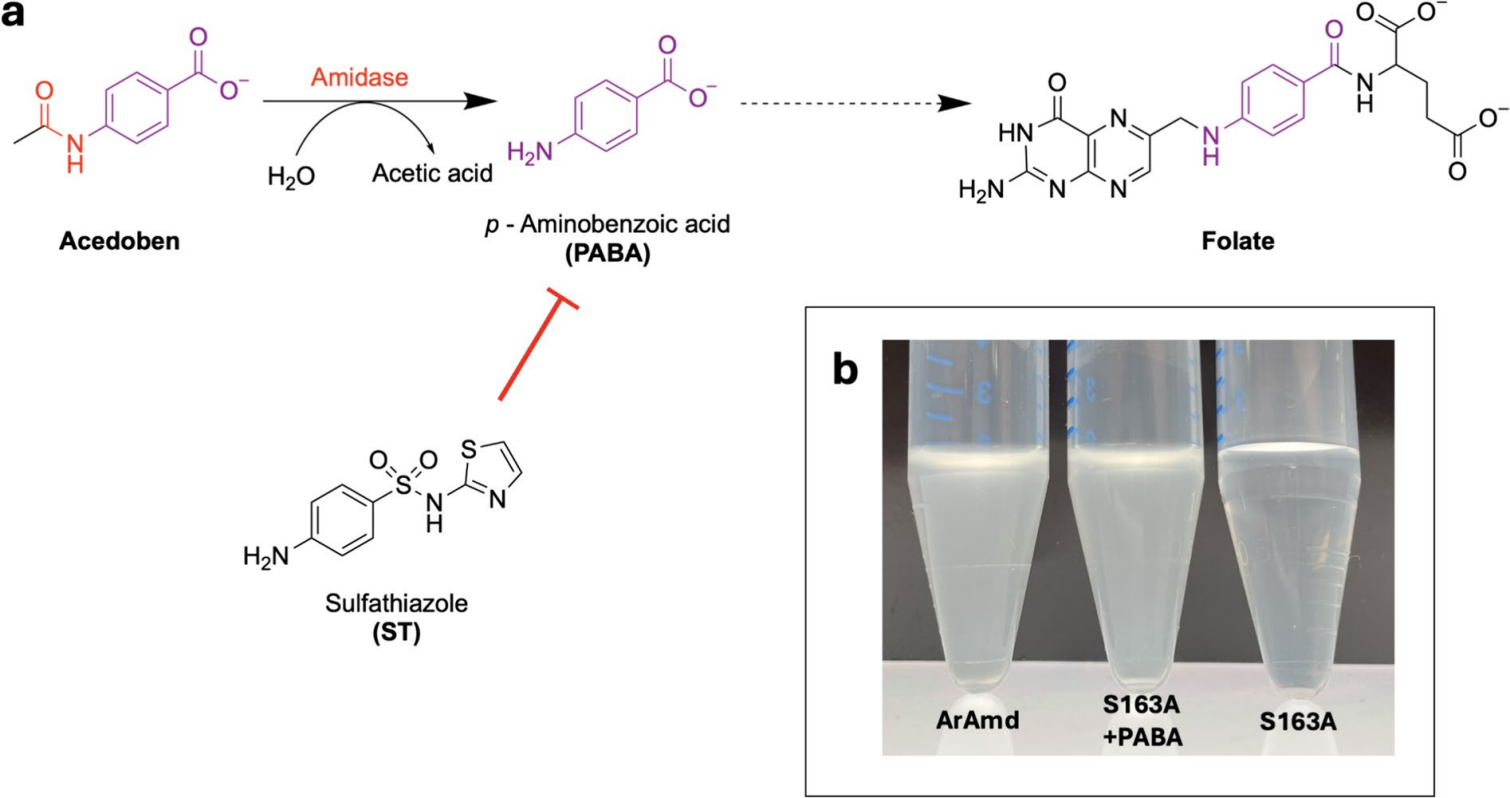
(**a**) The concept of the PABA growth selection assay for amidases. Acedoben, an amide-protected (red) derivative of *p*-aminobenzoic acid (PABA), can be deprotected by an amidase, resulting in the formation of PABA. Subsequently, PABA is available for folate synthesis, allowing PABA auxotrophs to grow. Sulfathiazole (ST), an antimetabolite of PABA, can be applied to fine-tune assay sensitivity. (**b**) Proof-of-concept for the PABA-growth selection assay. *E. coli_∆pabA*_pBAD_*aramd* (aryl acylamidase, ArAmd) served as positive control for the hydrolysis of acedoben to PABA. The inactive ArAmd_S163A served as negative control. Cells of pre-cultures grown in LB medium were washed and resuspended in screening medium and used to inoculate 2 mL screening medium containing kanamycin and ampicillin at a 1:100,000 dilution. The cultures were incubated at 37 °C, 200 rpm for 48 h. Cells expressing ArAmd showed significant growth, whereas the inactive variant could only grow when 5 μM PABA (as the equivalent to the 5 μM acedoben used) was added

Motivated by the goal to develop a highly sensitive, ultra-high throughput growth selection assay for amidase activity, we explored selection alternatives besides carbon or nitrogen sources. *p*-Aminobenzoic acid (PABA) is a key intermediate in the synthesis of folate, and concentrations as low as 1 nM in the growth medium are sufficient for *E. coli* PABA auxotrophs to grow, as demonstrated in our work (Fig. 1a). Folates act as coenzymes in several metabolic pathways leading to methionine, pantothenate, purines, and deoxythymidine monophosphate. They are composed of a pterin moiety, a PABA unit, and a γ-linked glutamate chain (Green and Matthews 2007). In *E. coli*, which harbors a complete folate biosynthesis pathway, PABA is synthesized from chorismate by the activity of three genes: *pabA, pabB, and pabC* (Anderson et al. 1991)*.* The PabAB heterodimer is responsible for the conversion of chorismate to the intermediate 4-amino-4-deoxychorismate, while PabC, an aminodeoxychorismate lyase, catalyzes the conversion of 4-amino-4-deoxychorismate to PABA (Huang and Pittard 1967; Huang and Gibson 1970; Nichols et al. 1989). *E. coli* cannot take up additional folate from the medium. However, the unstable folic acid decomposes to *p*-aminobenzoate-glutamate (PABA-glu) and PABA. While *E. coli* can take up PABA-glu via the transporter AbgT, PABA was shown to be membrane permeable (Carter et al. 2007; Maynard et al. 2018). The two PABA auxotrophs, *ΔpabA* and *ΔpabB*, of *E. coli* K-12 are part of the Keio collection (Baba et al. 2006) and were shown to require as little as 10 nM PABA for growth to saturation in minimal media. Further, it was demonstrated that these auxotrophs could be rescued by the chemical conversion of allyloxycarbonyl-protected PABA (alloc-PABA) to PABA in the presence of a ruthenium catalyst (Lee et al. 2013).

Inspired by these findings, herein, the growth of the auxotroph *E. coli* K-12 *ΔpabA* was coupled to the enzymatic production of PABA from the amide-protected precursor *N*-acetyl PABA (acedoben) so that promiscuous amidase activities of variants in mutant libraries can be detected with exceptional sensitivity. However, since a growth selection that requires only 1 nM of product would be very limited when it comes to further improving the activities of the initial variants, additionally, a strategy for decreasing the sensitivity of the assay was developed. It was shown that the sensitivity of the assay is adjustable by applying sulfonamide antibiotics, like sulfathiazole (ST), which are competitive inhibitors of dihydropteroate synthase, the enzyme that initiates the conversion of PABA to folate (Strauss et al. 1941; Rose and Fox 1942).

## Materials and Methods

All chemicals and reagents used, unless otherwise specified, were purchased from Sigma-Aldrich (St. Louis, MO, USA), Thermo Fisher Scientific (Waltham, MA, USA), Honeywell Fluka™ (Morristown, NJ, USA), Carl Roth GmbH (Karlsruhe, Germany), or TCI Deutschland GmbH (Eschborn, Germany). Oligonucleotides were obtained from Eurofins Scientific (Luxembourg).

### Selection of the *E. coli* K-12 *∆pabA* strain for growth assay establishment and minimal medium composition

The defined M9 glycerol minimal medium used by Lee et al. (Lee et al. 2013) contained 18.69 mM ammonium chloride, which was within tolerance, since the conditional auxotroph *E. coli* K-12 *ΔpabA*, referred to as *∆pabA* in the following, can grow in media with high ammonium concentrations (at least 100 mM) but not in media with low ammonium concentrations (Rayl et al. 1996). Further, it was clearly demonstrated that at least 10 nM PABA was required for the growth of the *∆pabA* strain, with no growth in the absence of added PABA. No such data were reported for the *∆pabB* strain. The *ΔpabA* strain also grew slightly faster than *ΔpabB* in the M9 medium supplemented with 10 μM PABA (Lee et al. 2013). Therefore, the *∆pabA* strain was selected in this work.

The defined M9 minimal medium was prepared according to Lee et al. (Lee et al. 2013). 5× M9 Minimal Salts (Sigma-Aldrich, catalog no. M6030, Merck, Darmstadt, Germany) were used as the base for the medium to avoid contamination with PABA. The final 1× M9 medium contained: 22 mM KH_2_PO_4_, 8.6 mM NaCl, 47.8 mM Na_2_HPO_4_, 18.7 mM NH_4_Cl, 1× trace elements (130 μM EDTA, 31 μM FeCl_3_*6 H_2_O, 6.2 μM ZnCl_2_, 760 nM CuSO_4_, 420 nM CoCl_2_, 1.62 μM H_3_BO_3_, and 81 nM MnCl_2_), 2 mM MgSO_4_, 100 μM CaCl_2_, 0.5% (v/v) glycerol as carbon source, 50 μg mL^−1^ kanamycin sulfate (to prevent contamination of the *∆pabA* strain with wild-type *E. coli*, since strains from the Keio collection are kanamycin resistant), and 5 μM *N*-acetyl PABA (acedoben). Acedoben was purified as described below. This medium, referred to as screening medium in the following, was used for the preparation of both liquid cultures or agar plates. Different concentrations of sulfathiazole (ST) were added as a competitive suppressor of PABA (Strauss et al. 1941; Rose and Fox 1942) as indicated. For the selection of transformants, 100 μg mL^−1^ ampicillin or 34 μg mL^−1^ chloramphenicol were added (for pBAD or pACYC vectors, respectively).

For screening plates, ~ 1.5% (w/w) agar-agar (catalog no. 5210.4, Carl Roth, Karlsruhe, Germany) was used. It was essential to wash the agar prior to use due to PABA contamination. Agar of higher purity (Noble Agar, catalog no. J10907-36, Thermo Scientific, Waltham, MS, USA) did not solve the problem of contamination sufficiently (data not shown). For washing, a 5× concentrated agar solution (75 g L^−1^) was autoclaved and solidified. The agar gel was chopped into small pieces using a spoon and washed three times 10 L ddH_2_O (ultra-pure water purified with Thermo Scientific™ Barnstead™ GenPure™ xCAD Plus, Thermo Fisher Scientific, Waltham, MA, USA) under stirring at 4 °C, changing the water once or twice a day. The washed agar was weighed, divided into portions sufficient for preparing 400 mL of screening agar each, and frozen at − 20 °C. The preparation scheme for fresh screening plates is given in Table S1.

### Preparation of Chemically Competent Cells of *ΔpabA* and Transformation by Heat-Shock

The protocol used for the preparation of chemically competent cells was adapted from the Inoue/Hanahan method (Floor 2019) and resulted in transformation efficiencies of ~ 10^8^ CFUs (μg DNA)^−1^, which was sufficient for assay preparation. The competent cells were dispensed into 50 μL aliquots and stored at − 80 °C.

For transformation, chemically competent cells were retrieved from − 80 °C and thawed on ice. Different amounts of DNA were added to a 50 μL cell aliquot, not exceeding a total volume of 5 μL DNA solution. The cell suspension was mixed by flicking the reaction tube several times, followed by 30 min incubation on ice. The cell suspension was heat-shocked at 42 °C for 45 s and put back on ice for 2 min before the addition of 950 μL of SOC medium (Cold Spring Harbor Protocols 2018). It was incubated at 37 °C with shaking at 200 rpm for 1 h. For estimation of the number of transformants/library size or the selection of transformants, 50 μL of the resulting culture was plated out on LB agar plates containing the appropriate antibiotics.

### Inoculation and Cultivation of Transformants

For inoculation, the cells of a 2 mL overnight culture grown in lysogeny broth (LB) medium (LB broth, Miller, catalog no. L3152-1KG, Sigma Aldrich, St. Louis, MO, USA) were sedimented, washed with 2 mL of screening medium, and resuspended in 2 mL of screening medium. After normalizing to OD_600_ = 1.0, the culture was diluted ~ 1:100,000 into 2 mL of screening medium in two steps of 6 μL into 2 mL or serially diluted and spread on screening agar plates. Especially for liquid cultures, high dilutions were important, because PABA-auxotrophic cells can divide up to seven times using intracellular PABA from the previous cultivation in a rich medium (Rose and Fox 1942). This means that cultures containing 50,000 cells mL^−1^ or more become turbid even in the absence of PABA (data not shown). Cultures were cultivated at 37 °C for ~ 48 h. Growth that took significantly longer than 48 h was categorized as false positive.

### Purification of Acedoben via Silica Column

Due to the extraordinary sensitivity of the assay, it was inevitable to purify the screening substrate to fall below contamination of PABA or other folate metabolites of < 0.02%. Acedoben (1 g) was dissolved in ~ 80 mL MeOH and ~ 3 g of Dicalite^TM^ speed plus (catalog no. 123380010, Thermo Scientific, Waltham, MA, USA) was added. The suspension was dried in a rotary evaporator. A column was loaded with ~ 100 g silica gel, homogenized in a 1:1-solvent mixture of petroleum ether (PE) and ethyl acetate (EA). The silica column was topped with 2–3 cm of sand before adding the acedoben/Dicalite^TM^ mixture. The column was successively washed with ~ 500 mL of PE/EA (1:1), ~ 500 mL of PE/EA (1:2), ~ 300 mL of PE/EA (1:4), and ~ 300 mL of pure EA, while samples of the flowthrough were investigated for PABA and acedoben content by thin layer chromatography (TLC, see below). Acedoben started to show up in the 100% EA fraction. Subsequently, acedoben was eluted with ~ 200 mL of 10% MeOH in EA (discarded), and 1 L of 10% MeOH in EA was collected. The acedoben was recovered by removing the solvents using a rotary evaporator and dried using a lyophilizer. Subsequently, the fluorescamine assay was used to determine residual PABA in the purified acedoben. The fluorescamine assay is a very sensitive fluorescence assay for amines (Udenfriend et al. 1972), which can be optimized for aromatic amines, like PABA, by a low pH in which aliphatic amines are protonated and therefore unreactive. For the assay, a 10× assay solution was freshly prepared containing 1 mM fluorescamine and 20% (v/v) glacial acetic acid in dimethyl sulfoxide (DMSO). In a microtiter plate, 20 μL substrate in DMSO (acedoben with potential PABA contamination, usually 1–10 mM acedoben) was mixed with 160 μL ddH_2_O before 20 μL of the 10× assay solution was added and mixed. The fluorescence was detected at 390 nm excitation and 475 nm emission.

### Enzyme Activity Screening by Thin Layer Chromatography (TLC) and Photometric Measurements

For *in vitro* enzyme activity screening in a microtiter plate (UV compatible), 20 μL of 1 mM acedoben in DMSO was added to 160 μL 50 mM phosphate buffer (pH 8), followed by 20 μL purified enzyme solution at different concentrations (for purification method see “Supplementary Information” section). The kinetics were measured spectrophotometrically in an MTP reader (BioTek Synergy H1, Agilent Technologies, Santa Clara, CA, USA) at a wavelength of 290 nm over 5 min with measurements taken every 30 s. The quantification was done with a calibration curve of 0–100 μM PABA. For TLC, the same reactions were done in a volume of 1 mL with 1 mM acedoben over a few hours. The reactions were quenched with EA for protein precipitation and the aqueous phase was loaded on a TLC plate with fluorescence indicator (ALUGRAM^®^ SIL G/UV_254_, catalog no. N729.1, Macherey-Nagel, Düren, Germany) and EA was used as mobile phase. The TLC plate was inspected under UV light (example given in Figure S3).

### Strains and Genes

The *E. coli* K12 auxotrophs *ΔpabA* (JW3323-KC; CGSC Strain #10483) and *ΔpabB* (JW1801-KC; CGSC Strain #9507) from the *E. coli* K-12 Keio Collection (Baba et al. 2006) were ordered from The National Bioresource Project *E. coli* Strain Office at the National Institute of Genetics in Mishima, Japan (https://shigen.nig.ac.jp/ecoli/strain/). The strains were received on small pieces of filter paper. The pieces of paper were added to a 5 mL LB medium containing 50 μg mL^−1^ kanamycin sulfate and incubated overnight at 30 °C. Glycerol stocks were prepared by adding 750 μL culture to 750 μL sterile 50% (v/v) glycerol before storage at − 80 °C. All constructed strains are listed in Table 1.

**Table 1:** Strains constructed within this work. For all strains the deletion strain *∆pabA* from the Keio collection (Baba et al. 2006), a conditional auxotroph for PABA in which the gene encoding the glutaminase subunit of aminodeoxychorismate synthase was deleted from the genome, was used. Genes were cloned into the ORF of the plasmids pBAD (*P*_*BAD*_ promoter) or pAC_s_ (*P*_*J23100*_ promoter)

Constructed strains	Description
*∆pabA*_pBAD_*pabA*	Positive control for the deletion strain *∆pabA*. The gene *pabA* was provided in a pBAD vector.
*∆pabA*_pBAD_*empty*	Negative control for the deletion strain *∆pabA*. The transformed pBAD vector did not contain an additional gene.
*∆pabA*_pBAD_*aramd*	Positive control for the amidase assay. The gene encoding ArAmd, which can hydrolyze acedoben, was introduced to the deletion strain.
*∆pabA*_pBAD_*aramd_*S163A	Negative control for the amidase assay. The gene encoding an inactive variant of ArAmd was introduced into the deletion strain.
*∆pabA*_pBAD_*umg-sp-2*	Positive control for the amidase assay with weaker activity towards acedoben compared to *∆pabA*_pBAD_*aramd.*
*∆pabA*_pAC_s__*aramd*	Variation of *∆pabA*_pBAD_*aramd* with stronger expression of ArAmd due to the constitutive promoter in pAC_s_.
*∆pabA*_pAC_s__*umg*-*sp-2*	Variation of *∆pabA*_pBAD_*umg*-*sp-2* with stronger expression of UMG-SP-2 due to the constitutive promoter in pAC_s_.

The DNA and protein sequences of the enzymes investigated in this study are given in the Supplementary Information. Aryl acylamidase (short: ArAmd, GenBank: ACP39716.2), UMG-SP-2 (GenBank: OP972510), aminodeoxychorismate synthase component 2 (short: PabA; Sequence ID: WP_220413299.1), and aminodeoxychorismate synthase component 1 (short: PabB; Sequence: WP_000854958.1) were used and a C-terminal His-tag was added.

### Cloning of Plasmid Constructs

All constructs were cloned using Gibson Assembly following the manual of the NEBuilder HiFi DNA Assembly Mix (catalog no. E2621S, New England Biolabs, Ipswich, MA, USA), if not mentioned otherwise. Primers were designed using the online tool NEBuilder by New England Biolabs (https://nebuilder.neb.com). Cloned constructs were verified by Sanger Sequencing (Microsynth, Balgach, Switzerland). The oligonucleotides used are given in the Supplementary Information in Table S2.

## Results

### Proof of Concept for the PABA Growth Selection Assay

First, the PABA auxotrophy of *∆pabA* as demonstrated by Lee et al. (Lee et al. 2013) was verified through the positive control *∆pabA*_pBAD_*pabA* and the negative control *∆pabA*_pBAD_*empty.* The minimal medium was supplemented either with 5 nM PABA, 5 μM acedoben (standard screening medium), or neither of the two (Figure S1). As expected, *∆pabA*_pBAD_*pabA* could grow in all media, whereas *∆pabA*_pBAD_*empty* only grew in a medium containing 5 nM PABA. This result verified that the strain cannot grow without PABA and that the applied acedoben was sufficiently pure. Next, to validate the concept of the assay, the aryl acylamidase ArAmd was introduced. Additionally, out of the recently published metagenomic urethanases UMG-SP-1-3 (Branson et al. 2023), the most thoroughly investigated UMG-SP-2 was chosen to be tested for its amidase activity towards acedoben. Both enzymes were expressed and purified (Figure S2, methods described in Supplementary Information) and exhibited amidase activity towards acedoben (Figure S3) which was ~ 34-fold lower for UMG-SP-2 (0.2278 U/mg ± 0.0194) compared to ArAmd (8.0491 U/mg ± 0.3710) in 50 mM phosphate buffer, pH 8. ArAmd was chosen as a positive control. As a negative control, the inactive variant ArAmd_S163A (both genes ordered in pBAD from BioCat, Heidelberg, Germany) with the catalytic serine substituted by an alanine, was used. The pBAD vector contains the *P*_*BAD*_ promoter, which is reported to be tightly controlled, therefore, it allows for weak induction to minimize stress by heterologous protein production under growth conditions in a minimal medium (Guzman et al. 1995). Subsequently, using a liquid medium, it was demonstrated that *∆pabA*_pBAD_*aramd* could grow in the defined screening medium, whereas *∆pabA*_pBAD_*aramd*_S163A could only grow when PABA was added to the culture (Fig. 1b). This demonstrated that not only PABA but also acedoben could enter the cell and that the assay setup was functional. The experiment suggested an optimal cultivation time of ~ 48 h at 37 °C to obtain significant growth. Despite the tight induction system, preliminary experiments indicated that the basal expression in the absence of the inducer arabinose was enough for the cells containing pBAD_*aramd* to grow; arabinose induction did not further increase growth (data not shown). These results were later confirmed by experiments with *∆pabA*_pBAD_*umg-sp-2* (see below).

### Assay Adaptation to an Agar Plate Format

**Fig. 2:**
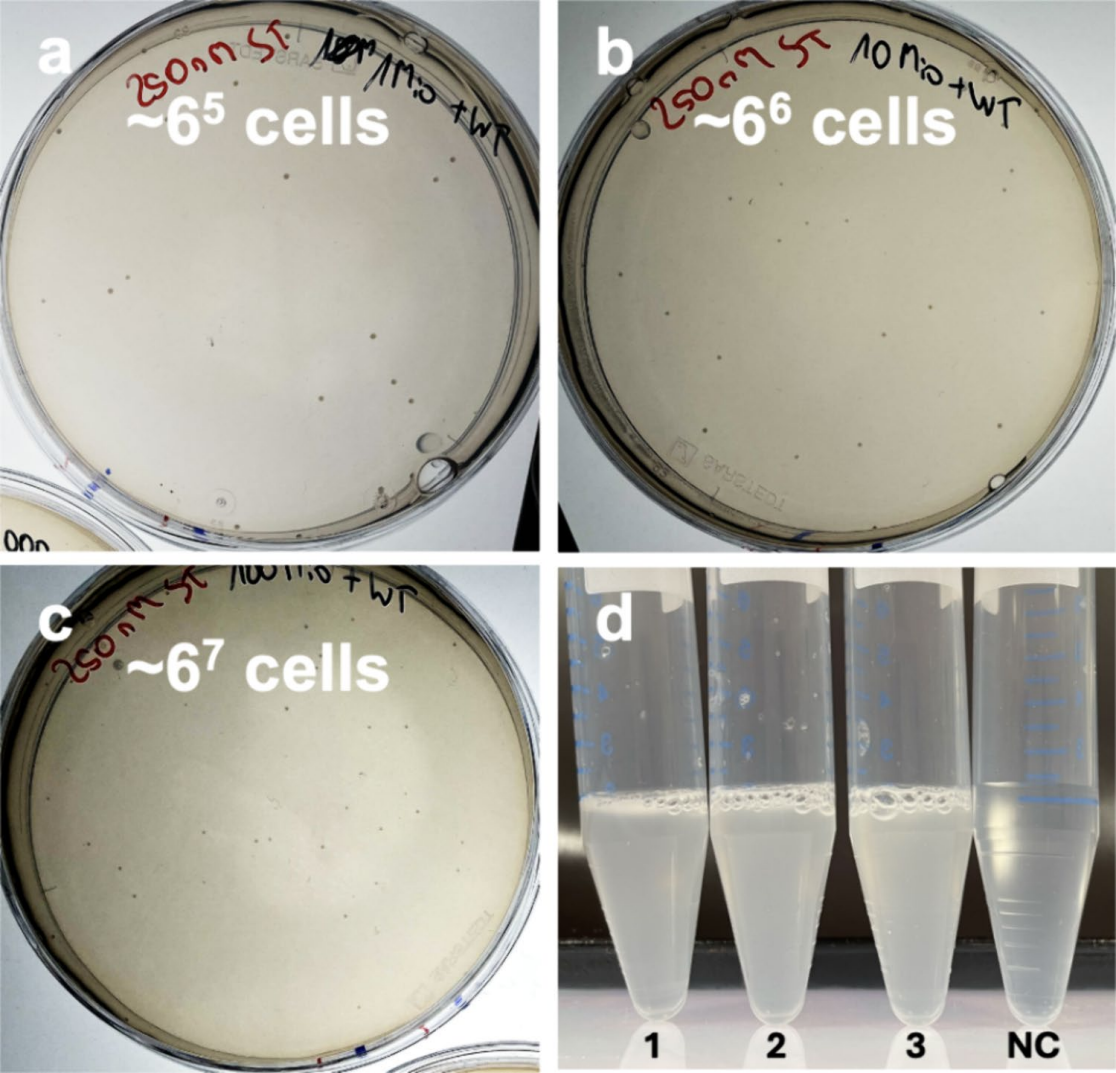
Assay adaption to the agar plate format. Screening plates containing 250 nM ST were inoculated to evaluate the assay's performance across varying cell densities. The figure shows plates after incubation at 37 °C for 48 h with differing numbers of control cells: (**a**) ~ 600,000 negative control cells (*E. coli*_*∆pabA*_pBAD_*aramd*_S163A) and about 30 positive control cells (*E. coli*_*∆pabA*_pBAD_*aramd)*, (**b**) ~ 6 million negative control cells and 30 positive control cells, and (**c**) ~ 60 million negative control cells and 30 positive control cells. Despite the vast differences in background cell density, each plate consistently showed growth of only the positive control colonies, confirming the assay's specificity. (**d**) detail of the subsequent growth validation in liquid screening medium of three colonies (labeled 1–3) transferred from the plate shown in (**c**), a no-colony (NC) sample scraped from the plate without touching any colonies. Only the transferred colonies demonstrated growth, further verifying the accuracy of the assay in distinguishing positive controls under varying experimental conditions. The grown liquid cultures were verified to consist of *E. coli_∆pabA*_pBAD_*aramd* and not *E. coli*_*∆pabA*_pBAD_*aramd*_S163A by Sanger sequencing

To increase throughput by better hit separation and to minimize the rate of false positives due to diffusion of PABA from active to inactive cells, the assay was transferred to agar plates. The agar was pre-treated to reduce folate metabolites (e.g., PABA) as contaminants present in commercial supplies as described in detail in the “Method” section. Although pre-treatment significantly reduced background growth, ST, a sulfonamide known to be a potent competitive suppressor of PABA (Strauss et al. 1941), was applied at a concentration of 250 nM to fully suppress the growth of the negative control. To estimate the applicability of the optimized setup, the maximal cell density of the negative control (*ΔpabA*_pBAD_*aramd*_S163A) was evaluated, because too high cell densities would lead to growth independent of amidase activity due to traces of intracellular folate metabolites as described above (Rose and Fox 1942). Therefore, an overnight culture of the negative control in LB medium supplemented with kanamycin and ampicillin was cultivated, and the cells were washed and resuspended in the screening medium, normalizing the culture to OD_600_ = 1.0. Subsequently, 100 μL of different dilutions of the normalized culture on both screening plates (plates with washed agar and screening medium) and control plates containing LB medium and 1.5% commercial agar to estimate the number of cells were plated out (Figure S2). Growth on the LB plate controls suggested a cell density of the OD_600_ = 1.0 cultures of ~ 6^9^ CFU mL^−1^. Thus, it was concluded that cell densities exceeding 6 million cells per plate of the *Δpab*_pBAD_*aramd*_S163A as negative control did not grow on the screening plates with 250 nM ST. Finally, the experiment was repeated by adding a strong dilution of a culture of *Δpab*_pBAD_*aramd* as a positive control to obtain ~ 10–50 cells per plate to different dilutions of the negative control (Fig. 2a–c). LB plates (example given in Figure S4c) revealed a cell density of ~ 600,000 CFU (Fig. 2a), 6 million CFU (Fig. 2b), and 60 million CFU (Fig. 2c) of the negative control per screening plate. The size of the PC colonies decreased with increasing cell density of the negative control indicating that the sensitivity of the assay decreased with higher cell loading. However, colonies growing on these plates were taken and resuspended in a liquid screening medium and all cultures grew after 48 h at 37 °C (Fig. 2d). Sequencing confirmed the presence of the positive-control plasmid. Therefore, we could demonstrate that a single positive variant was found in ~ 60 million negative clones on a single screening plate.

### Comparison of Different Amidases and Adjustment of Assay Sensitivity

**Fig. 3:**
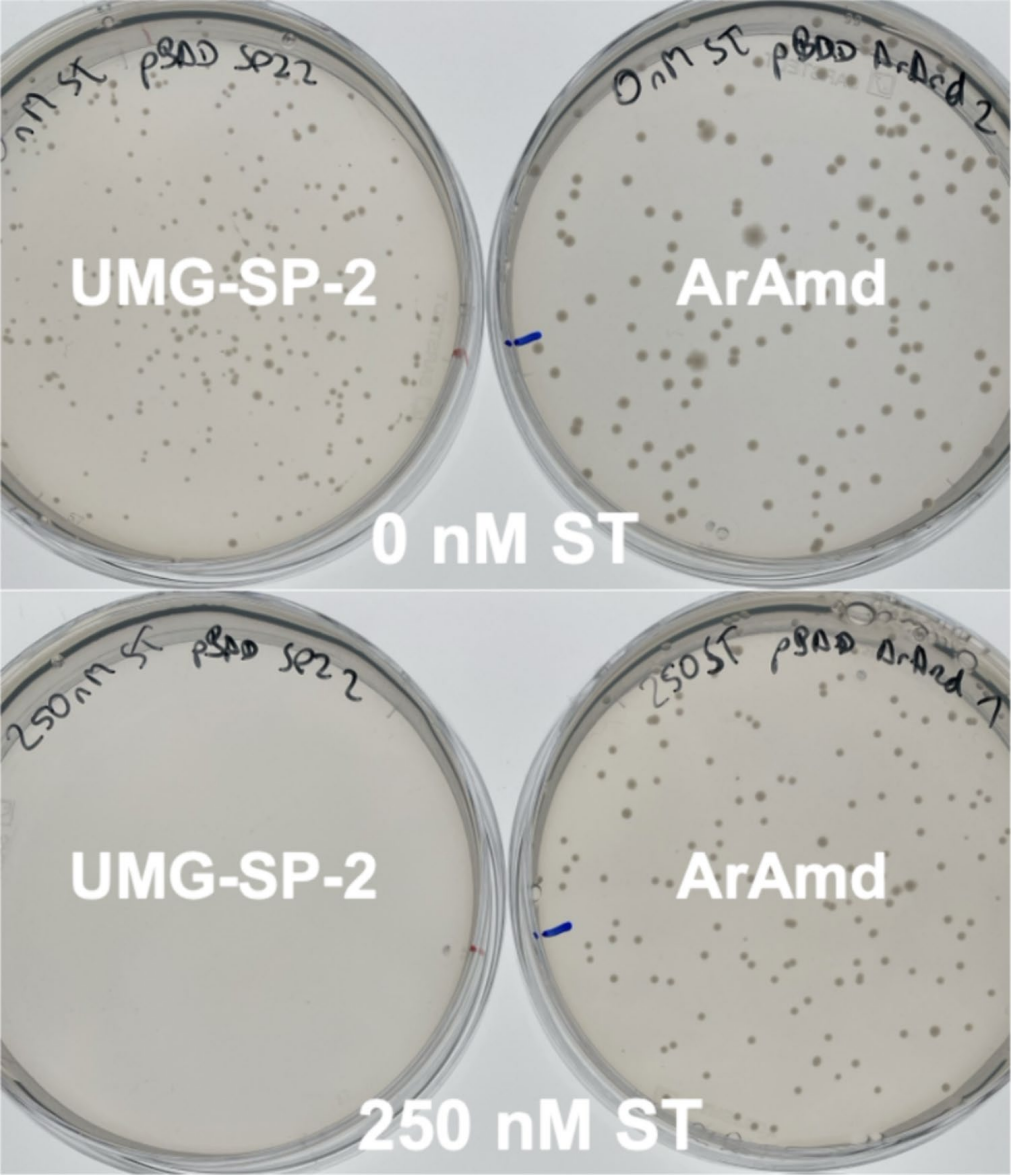
Screening plates of *∆pabA*_pBAD_*umg*-*sp-2* and *∆pabA*_pBAD_*aramd* supplemented with ampicillin and either 0 nM ST or 250 nM ST. Without ST both strains developed strong colonies with a visible difference in size between the UMG-SP-2 strain and the ArAmd strain. With 250 nM ST, only the ArAmd strain developed colonies which did not significantly lose size compared to 0 nM ST. The plates were inoculated with 100 μL of a 1:200,000-dilution of cells of an overnight culture washed and resuspended to OD_600_ = 1.0 in screening buffer. The experiment was done in duplicates

To compare ArAmd and UMG-SP-2 under assay conditions, the gene encoding UMG-SP-2 was cloned into the pBAD vector and the plasmid was transformed into *E. coli ∆pabA*. Cells of overnight cultures in LB medium of both strains supplemented with kanamycin and ampicillin, *∆pabA*_pBAD_*aramd* and *∆pabA*_pBAD_*umg*-*sp-2*, were washed and resuspended to an OD_600_ = 1.0 in screening buffer and subsequently diluted to obtain ~ 100–300 CFUs per plate before spreading them on screening plates with either 0 nM ST or 250 nM ST (Fig. 3). Without the competitive suppressor, both strains developed strong colonies at 37 °C after 48 h, whereas the ArAmd-strain developed bigger colonies compared to the UMG-SP-2-strain. However, already 250 nM ST was enough to completely suppress the growth of the UMG-SP-2-strain, whereas the size of the ArAmd-colonies barely changed.

Under experimental conditions, *∆pabA*_pBAD_*aramd,* and *∆pabA*_pBAD_*umg*-*sp-2* showed a significant difference in growth indicating a difference in the hydrolysis of acedoben and, thus, the production of PABA. Therefore, it was investigated, if the cell growth could be altered to tune the assay sensitivity for different amidases by changing the expression system. The assay was developed using the tight and uninduced *P*_*BAD*_ promoter for enzyme expression. Although preliminary experiments suggested no or negative impact of arabinose induction on the *P*_*BAD*_ promoter, it was investigated whether induction with 0.05% arabinose would increase the assay’s sensitivity for UMG-SP-2 (Table 2). At 0 nM ST, the strain developed big colonies without arabinose, while the colonies were significantly smaller with 0.05% arabinose. At 250 nM ST and 500 nM ST, the strain developed no colonies without arabinose and barely visible colonies with 0.05% arabinose. There was no growth at 1 μM ST. Although the cells could withstand higher concentrations of ST with 0.05% arabinose compared to no arabinose, they developed bigger colonies without arabinose when no ST was applied. Additionally, the colonies with 0.05% arabinose stayed relatively small even after several days of incubation at 37 °C.

**Table 2:** Growth of *∆pabA*_pBAD_*umg*-*sp-2* on screening plates with different concentrations of ST and either no arabinose or 0.05% arabinose. The plates were incubated for 48 h at 37 °C

ST conc.	0 nM	250 nM	500 nM	1 μM
No arabinose	Big colonies	No growth	No growth	No growth
0.05% arabinose	Small colonies	Barely visible colonies	Barely visible colonies	No growth

**Fig. 4:**
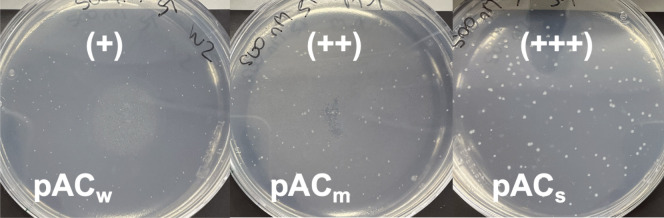
Screening plates containing chloramphenicol and 500 nM ST inoculated with *∆pabA* carrying pAC_w_*_umg-sp-2*, pAC_m__*umg*-*sp-2,* or pAC_s__*umg*-*sp-2*. The colonies clearly increased in size with the strength of the promoter. (+) indicates weakest growth and (+++) indicates strongest growth. The plates were inoculated with 100 μL of a 1:200,000-dilution of cells of an overnight culture washed and resuspended to OD_600_ = 1.0 in screening buffer. The experiment was done in duplicates

Although the results of the arabinose induction were ambiguous, it could be concluded that the assay sensitivity could not be increased significantly by arabinose induction. Alternatively, constitutive expression was investigated. Using a pACYC-derived vector (pA15 origin of replication, ~ 10–12 copies, chloramphenicol resistance) containing different variants of the constitutive promoter *P*_*J23100*_ instead of a lactose-inducible *P*_*T7*_ promoter (Anderson 2006), the assay sensitivity for UMG-SP-2 was further investigated. Herein, the plasmids were named pAC_w_ (weak expression level, *P*_*J23116*_ ≈ 0.16 *P*_*J23100*_), pAC_m_ (medium expression level, *P*_*J23106*_ ≈ 0.45 *P*_*J23100*_), and pAC_s_ (strong expression level, *P*_*J23100*_), as also defined in previous works (Anderson 2006; Wu et al. 2022). The three different promoter variants were compared on screening plates containing chloramphenicol and 500 nM ST (Fig. 4). An obvious increase in colony size with increasing promoter strength was visible. Therefore, for further experiments only pAC_s_ were investigated.

Finally, the impact of different concentrations of ST was investigated. Screening plates with several concentrations between 0 nM and 1 μM ST were inoculated with *∆pabA* containing either pBAD_*umg*-*sp-2*, pBAD_*aramd*, pAC_s__*umg*-*sp-2*, or pAC_s__*aramd* (Table 3; plates shown in Figure S5). The plates inoculated with the strains containing the pBAD-vectors repeated the trend of Fig. 4. While *∆pabA*_pBAD_*umg*-*sp-2* did not grow at 250 nM ST or higher, *∆pabA*_pBAD_*aramd* developed colonies even at 1 μM ST with decreasing colony size from 0 nM ST to 1 μM ST. Further, as demonstrated above, the sensitivity for UMG-SP-2 could be drastically increased by exchanging pBAD with pAC_s_, showing similar and even slightly better growth compared to the strain containing pBAD_*aramd*. ArAmd, however, did not follow this trend. Colonies of *∆pabA*_pAC_s__*aramd* on screening plates containing 0–750 nM ST were smaller compared to the strain containing pBAD_*aramd* and no colonies were observed at 1 μM ST. Hence, stronger expression through the constitutive promoter *P*_*J23100*_ compared to the *P*_*BAD*_ promoter was beneficial only for the selection of UMG-SP-2. The amidase ArAmd profited from the low basal expression of the *P*_*BAD*_ promoter.

**Table 3:** Growth of different strains on screening plates with increasing concentrations of ST. Colony growth on the plates was observed in relation to the other plates. (–) displays no growth, (+) displays lowest observed growth, and (++++) displays highest observed growth. Whereas the pAC_s_-vector significantly increased the assay sensitivity for the less active UMG-SP-2 compared to the pBAD-vector, this was not the case for the more active ArAmd, where the sensitivity even slightly decreased. The plates were inoculated with 100 μL of a 1:200,000 dilution of cells of an overnight culture, washed and resuspended to OD_600_=1.0 in screening buffer. The experiment was done in duplicates

ST conc.	0 nM	250 nM	750 nM	1 μM
pBAD_*umg*-*sp-2*	(+++)	(–)	(–)	(–)
pAC_s__*umg*-*sp-2*	(++++)	(++++)	(+++)	(++)
pBAD_*aramd*	(++++)	(++++)	(++)	(+)
pAC_s__*aramd*	(++++)	(+++)	(+)	(–)

Although the significant difference in specific activity towards acedoben between UMG-SP-2 and ArAmd may explain the difference in assay sensitivity for UMG-SP-2 changing the expression system from the pBAD vector to the pAC_s_ vector, it did not explain the negative impact of the stronger expression system on the sensitivity for ArAmd. Therefore, the impact of the expression rate of both enzymes in the pAC_s_ expression system on cell growth in both the screening medium (with or without additional PABA) and the LB medium was investigated. Indeed, significantly lower cell growth of *∆pabA*_pAC_s__*aramd* compared to *∆pabA*_pAC_s__*umg*-*sp-2* could be observed in liquid cultures of screening medium, and the addition of 5 μM PABA to the screening medium as an equivalent to the 5 μM acedoben used did not have any impact on the growth (Figure S6). Furthermore, the cell growth of both strains did not differ within 6.5 h when they were cultivated in LB medium (Figure S7). Tracking enzyme expression within the different cultures through sodium dodecyl sulfate – polyacrylamide gel electrophoresis (SDS-PAGE), it could be demonstrated that ArAmd was expressed at higher levels than UMG-SP-2 (Figure S8). Additionally, this was supported by the prediction of the translation initiation rates from the corresponding mRNAs (starting from the ribosome binding site (RBS) ending at the stop codon) of both enzymes for the RBS in pAC_s_ (UMG-SP-2: 4.48; ArAmd: 41.56) and pBAD (UMG-SP-2: 9.43; ArAmd: 111.03), calculated using the RBS calculator by De Novo DNA (https://www.denovodna.com) (Salis 2011). Therefore, it could be concluded that the lower cell growth of the higher active ArAmd compared to the lower active UMG-SP-2, when using the pAC_s_ expression system, was not caused by the amount of PABA produced through the activity of the enzyme, but rather by the stress of high expression levels in the minimal screening medium. Thus, we offer different, well-investigated expression systems as a potent variable for assay adaptation to desired engineering targets.

## Discussion

Engineering biocatalysts through directed evolution requires potent, sensitive, and fast screening methods. Herein, we introduce a growth selection assay based on the production of PABA, a precursor to the cofactor folate, which is suitable for detecting amidase activity. Compared to common growth selection assays based on nitrogen or carbon sources, the coupling of growth to an essential metabolite only needed in small quantities for proper metabolic function enables extraordinary sensitivity. It was demonstrated that PABA is only needed in amounts as low as 1 nM for the growth of an *E. coli* auxotroph (*∆pabA*). This assay is particularly valuable for challenging projects, such as those enhancing the low-level promiscuous amidase activity of esterases. Through the application of different expression systems, the sensitivity of the assay could be easily adjusted to the individual needs of the project. Complementary, the sensitivity could be fine-tuned by the application of increasing concentrations of the antimetabolite ST, which is convenient for iterative rounds of mutation in directed evolution, as they are usually applied. Next to ST, other sulfonamides, like sulfadiazine, sulfapyridine, sulfaguanidine, and sulfanilamide with decreasing suppression as investigated by Strauss et al. (Strauss et al. 1941), could add flexibility to fine-tuning the assay for individual purposes. Additionally, PABA-derived amides structurally mimic the broad class of commonly used chromogenic substrates derived from *p*-nitroaniline, suggesting that the assay should be broadly applicable to the identification of diverse amidases (Hemker 2012).

Compared to medium throughput methods like various chromatographic methods or high throughput strategies like microfluidics or cell sorting, the PABA-based growth selection assay can operate very large enzyme libraries solely limited by transformation efficiency in a convenient manner and without the usage of expensive and complex devices. As demonstrated here, using this assay, tens of millions of variants can be screened on only one single screening plate within two days of incubation. Additionally, the basis of this assay, protecting PABA with a reactive group, could easily be adjusted to reactive groups other than amides, increasing the applicability of this assay. While this assay promises applicability for directed evolution, by incorporating additional steps it can also be applied for the screening of metagenome libraries. For the latter application, false positive hits might be related to PabA homologs, complementing the gene deletion in the employed *∆pabA* strain. Subsequently, another round of the PABA growth selection assay can be applied, where the positive hits are replicated in two screening cultures without acedoben, where one contains PABA, and one does not. Hits that grow in both media are false positives (containing the *pabA* gene) because they can grow in a PABA-free medium without acedoben, whereas the hits that grow only in the medium containing PABA are correct positive hits. Alternatively, positive hits can easily be transferred to another assay, like a halo formation assay (Branson et al. 2022) or a microtiter plate-based assay (Henke and Bornscheuer 2003), increasing the throughput of these methods by far.

## Supplementary Information

ESM 1 Table S1. Preparation scheme for screening plates. It is crucial to cool down the autoclaved agar before adding M9 salts, because of oxidation which can occur at high temperatures. Figure S1. Liquid cultures of ∆pabA_pBAD_pabA and *∆pabA*_pBAD_empty in screening medium (a), screening medium containing 5 nM PABA (b), and plane minimal medium without acedoben and PABA (c). *∆pabA*_pBAD_pabA grew in all media, whereas *∆pabA*_pBAD_*empty* could only grow in medium containing 5 nM PABA. Figure S2. SDS-PAGE analysis of purified UMG-SP-2, ArAmd, and ArAmd_S163 used for in vitro experiments. M: Marker. Figure S3. Thin-layer chromatography of reaction products from the hydrolysis of acedoben using UMG-SP-2, ArAmd, and ArAmd_S163A together with the controls acedoben, PABA, and enzyme without substrate. While UMG-SP-2 and ArAmd fully converted acedoben to PABA, ArAmd_S163A did not. Fig. S3 Thin-layer chromatography of reaction products from the hydrolysis of acedoben using UMG-SP-2, ArAmd, and ArAmd_S163A together with the controls acedoben, PABA, and enzyme without substrate. While UMG-SP-2 and ArAmd fully converted acedoben to PABA, ArAmd_S163A did not. Figure S5. Screening plates containing chloramphenicol (pACs) or ampicillin (pBAD) and different concentrations of ST, inoculated with *∆pabA* carrying pBAD_*umg-sp-2* (upper left), pBAD_*aramd* (upper right), pACs_ *umg-sp-2* (lower left), or pACs_*aramd* (lower right). Whereas the pACs-vector significantly increased the assay sensitivity for UMG-SP-2 compared to the pBAD vector, this was not the case for ArAmd, where the sensitivity even slightly decreased. The plates were inoculated with 100 μL of a 1:200,000-dilution of an overnight culture washed and resuspended to OD_600_ = 1.0 in screening buffer. The experiment was done in duplicates. Figure S6. Growth of *∆pabA*_pACs_*aramd* and *∆pabA*_pACs_*umg-sp-2* in screening medium (M9) and screening medium with an additional 5 μM PABA (M9+PABA). The strain expressing UMG-SP-2 showed significantly stronger growth compared to the strain expressing ArAmd, whereas the addition of PABA did not have any impact for both strains. The cultures had a volume of 20 mL and were inoculated 1:100,000 with log-phase pre-cultures normalized to an OD_600_ of 0.57. Strains were cultivated in duplicates. Figure S7. Growth curve of *∆pabA*_pACs_*aramd* and *∆pabA*_pACs_*umg-sp-2* in LB medium. No difference in growth was visible between the strains within 6.5 h. The cultures had a volume of 30 mL and were inoculated 1:150 with log-phase pre-cultures normalized to an OD_600_ of 0.56. Strains were cultivated in duplicates. Figure S8. SDS-PAGE analysis of cell cultures of *∆pabA*_pACs_*aramd* and *∆pabA*_pACs_*umg-sp-2* under different cultures conditions. From left to right: Frist gel: Marker (M), cultures in LB (1, 2). Faint bands of ArAmd were visible, no bands of UMG-SP-2 were visible after 6.5 h of incubation. Second gel: Marker (M), ArAmd cultures in screening medium (M9) and screening medium with 5 μM additional PABA (M9+PABA) in duplets (1, 2), UMG-SP-2 cultures in screening medium (M9) and screening medium with 5 μM additional PABA (M9+PABA) in duplets (1, 2). Strong bands of ArAmd and no bands of UMG-SP-2 were visible after 42 h of incubation. No difference was visible between screening medium and screening medium with additional PABA. All cultures were normalized to OD_600_ = 2.0. Table S2: DNA sequences and oligonucleotides. Protein sequences of used enzymes. (PDF 4846 kb)

## Data Availability

The datasets generated during and/or analyzed during the current study are available from the corresponding author on reasonable request.
