# Supplementary material for: An Extremely Sensitive Ultra-High Throughput Growth Selection Assay for the Identification of Amidase Activity
Source: Appl Microbiol Biotechnol. 2024 Jun 24;108:392. doi: 10.1007/s00253-024-13233-z (PMC11194204; doi:10.1007/s00253-024-13233-z)
Supplement: Supplementary file 1 [file 253_2024_13233_MOESM1_ESM.pdf]

Supplementary Information

An Extremely Sensitive Ultrahigh Throughput Growth Selection Assay for the Identification of Amidase Activity

Yannick Branson[1], Bjarne Schnell[1], Celine Zurr[1], Thomas Bayer[1], Christoffel P.S. Badenhorst[1], Ren Wei[1], Uwe T. Bornscheuer[1]*

[1]Department of Biotechnology & Enzyme Catalysis, Institute of Biochemistry, University of Greifswald, Greifswald 17487, Germany

*Corresponding author: E-mail: uwe.bornscheuer@uni-greifswald.de

Content

Materials and Methods 4

Table S1 *Preparation scheme for screening plates.* 4

Purification of enzymes 4

Determination of specific activities 5

Additional Figures 6

Fig. S1 *Liquid cultures of ∆pabA_pBAD_pabA and ∆pabA_pBAD_empty in screening medium* 6

Fig. S2 *SDS-PAGE of purified UMG-SP-2, ArAmd, and ArAmd_S163 used for in vitro experiments. M: Marker.* 6

Fig. S3 *Thin-layer chromatography of reaction products from the hydrolysis of acedoben* 6

Fig. S4 *Dilution series of ∆pabA_pBAD_aramd_S163A on screening plates containing 250 nM ST.* 7

Fig S5 *Screening plates with chloramphenicol (pAC$_s$) or ampicillin (pBAD) and different concentrations of ST, inoculated with ∆pabA containing pBAD_umg-sp-2 (upper left), pBAD_aramd (upper right), pAC$_s$_ umg-sp-2 (lower left), or pAC$_s$_aramd (lower right).* 8

Fig S6 *Growth of ∆pabA_pAC$_s$_aramd and ∆pabA_pAC$_s$_umg-sp-2 in screening medium (M9) and screening medium with additional 5 µM PABA (M9+PABA).* 9

Fig S7 *Growth curve of ∆pabA_pAC$_s$_aramd and ∆pabA_pAC$_s$_umg-sp-2 in LB medium.* 9

Fig S8 *SDS-PAGE of cell cultures of ∆pabA_pAC$_s$_aramd and ∆pabA_pAC$_s$_umg-sp-2 in different cultures.* 10

DNA sequences and oligonucleotides 10

aramd 11

aramd_S163A 11

umg-sp-2 12

pabA 12

pabB 13

Protein sequences of used enzymes 13

ArAmd 13

ArAmd_S163A 13

UMG-SP-2 14

PabA 14

PabB 14

Reference **15**

Materials and Methods

Table S1 Preparation scheme for screening plates. It is crucial to cool down the autoclaved agar before adding M9 salts, because of oxidation which can occur at high temperatures.

Component	Volume	Final conc.
Washed agar for 400 mL final volume		1x
MilliQ	Fill to 240 mL	
	Autoclave; cool down to ~60°C	
5x M9 (warm)	60 mL	1x
$MgSO_4$ (1 M)	600 µL	2 mM
$CaCl_2$ (1 M)	30 µL	100 µM
Glycerol (50%)	3 mL	0.5%
100x Trace elements	3 mL	1x
Kan (50 mg mL^{-1})	300 µL	50 µg mL^{-1}
Amp (100 mg mL^{-1}) or Chl (34 mg mL^{-1})	300 µL	100 µg mL^{-1} or 34 µg mL^{-1}
ST (1 mM)	Variable	Variable
Acedoben (100 mM)	15 µL	5 µM

Purification of enzymes

Expression vectors were transformed into *E. coli* BL21(DE3) (pET-vector) or Δ*pabA* (pAC vector), and the cells were plated out on LB agar containing 50 µg mL^{-1} kanamycin or 34 µg mL^{-1} chloramphenicol. Single colonies were used to inoculate overnight cultures in LB medium containing the corresponding antibiotic. Overnight cultures (1 mL) were used to inoculate LB-5052 medium (200 mL containing corresponding antibiotic). Cultures were incubated at 37 °C for 4 h (shaking at 100 rpm for baffled flasks or 200 rpm for smooth flasks). The temperature was then reduced to 20 °C and the cultures were incubated for another 24 h. Cells were harvested by centrifugation for 20 min at 4,500 *g* and 4 °C. Cell pellets were stored at -20 °C before purification. Cells were resuspended and disrupted by sonication of the cell pellets in 4 mL lysis buffer (50 mM sodium phosphate buffer, pH 8.0, containing 300 mM sodium chloride and 10 mM imidazole) per 1 g of cell pellet. Three cycles of ultrasonication (2.5 min, 50% pulse, 50% power) were performed on ice using a SONOPULS HD 2070 (BANDELIN electronic GmbH & Co. KG). Lysates were clarified by centrifugation at 10,000 *g* and 4 °C for 60 min. The recombinant proteins were purified by IMAC. The clarified lysates were applied to ROTI®Garose His/Ni matrix (1 mL) that had been equilibrated with lysis buffer. Columns were washed with ten column volumes of wash buffer (50 mM sodium phosphate buffer, pH 8.0, containing 300 mM NaCl and 20 mM imidazole). The proteins were then eluted with 15 mL elution buffer (50 mM sodium phosphate buffer, pH 8.0, containing 300 mM sodium chloride and 250 mM imidazole). The eluates were concentrated to 2.5 mL using Vivaspin 20 ultrafiltration units (10 kDa

MWCO). Finally, the elution buffer was exchanged with storage buffer (50 mM sodium phosphate buffer, pH 8.0) using PD10 columns (GE Healthcare). The proteins were eluted from the PD10 columns using 3.5 mL of storage buffer. Proteins in storage buffer were stored on ice in a cold room and used within a few days.

Determination of specific activities

Specific activities were determined at 25 °C in 50 mM sodium phosphate buffer, pH 8.0, containing 100 µM acedoben. Acedoben was added from a 1 mM stock in DMSO (10% final concentration of DMSO). Purified amidases were diluted in storage buffer (50 mM sodium phosphate buffer, pH 8.0) prior to addition to the assay to give a linear increase in absorbance at 290 nm over 5 min. These diluted samples (20 µL) were pipetted (twice in triplicates in two separate experiments) into clear and UV-compatible 96 well plates (without lids). The reaction buffer was then pipetted into the wells using a multichannel pipette (final volume of 200 µL). Measurements were carried out in 96-well plates in a microtiter plate reader. Absorbance at 290 nm was measured every 30 s for 5 min. Specific activities (given in main text) were calculated from the linear increase in absorbance in the first few minutes and converted to µmol min^{-1} mg^{-1} or U mg^{-1} using a PABA calibration curve in the assay buffer. Since acedoben absorbs at 290 nm as well, the dilution series was pipetted starting from 0 µM PABA and 100 µM acedoben to 100 µM PABA and 0 µM acedoben. The absorbance at 290 nm was linear from 0 µM to 100 µM PABA.

Additional Figures

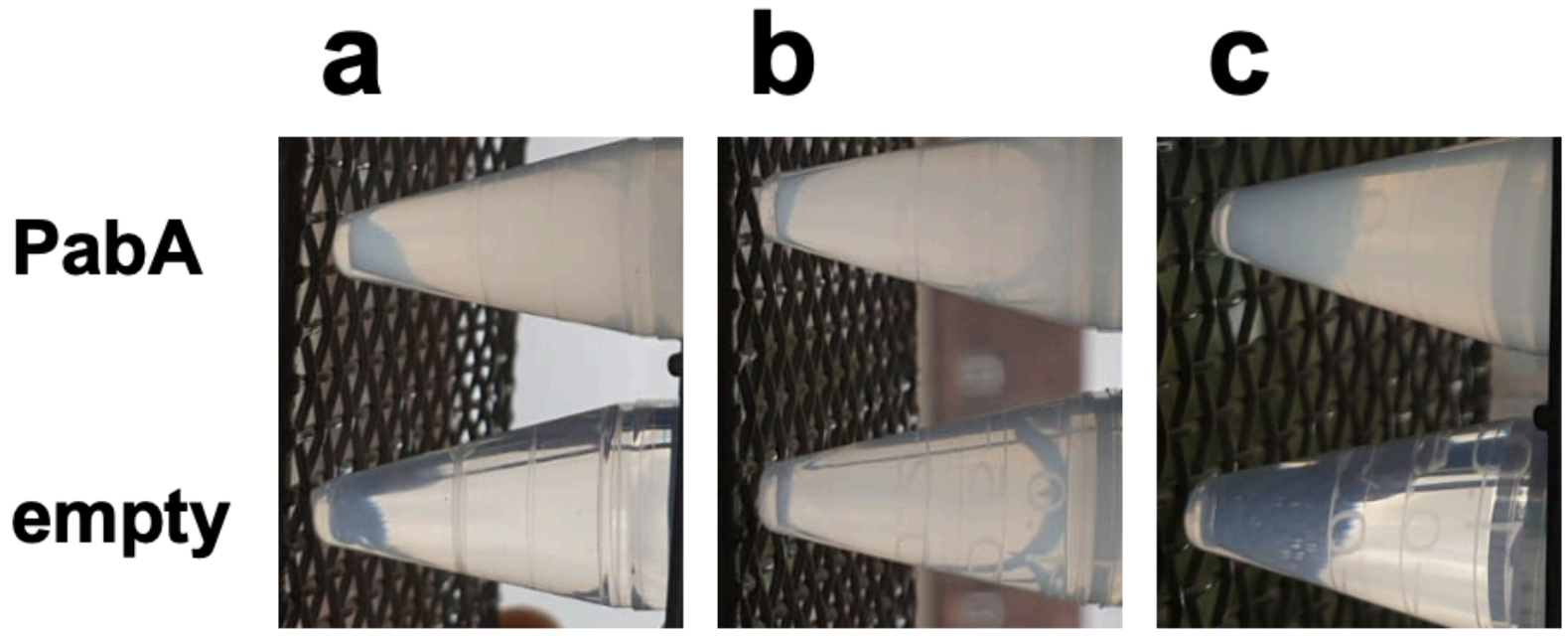

Fig. S1 Liquid cultures of Δ*pabA*_pBAD_*pabA* and Δ*pabA*_pBAD_empty in screening medium (**a**), screening medium containing 5 nM PABA (**b**), and plane minimal medium without acedoben and PABA (**c**). Δ*pabA*_pBAD_*pabA* grew in all media, whereas Δ*pabA*_pBAD_*empty* could only grow in medium containing 5 nM PABA.

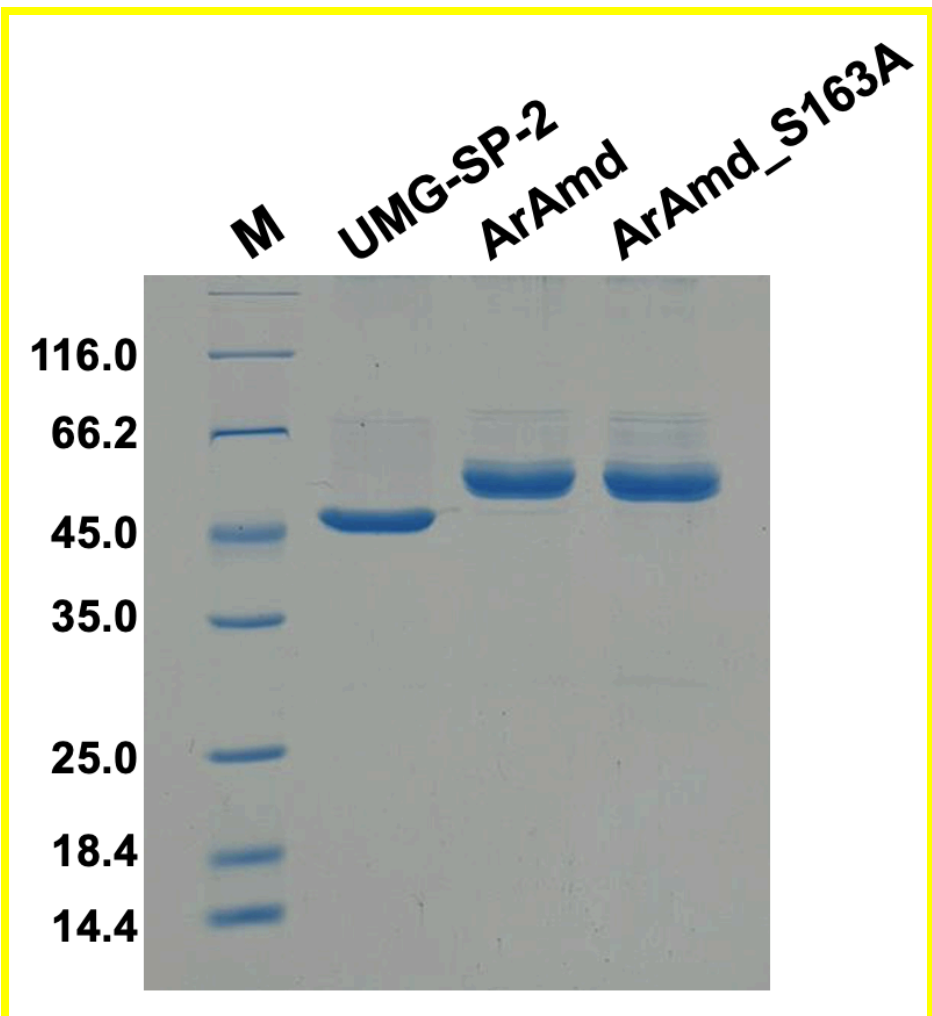

Fig. S2 SDS-PAGE analysis of purified UMG-SP-2, ArAmd, and ArAmd_S163 used for *in vitro* experiments. M: Marker.

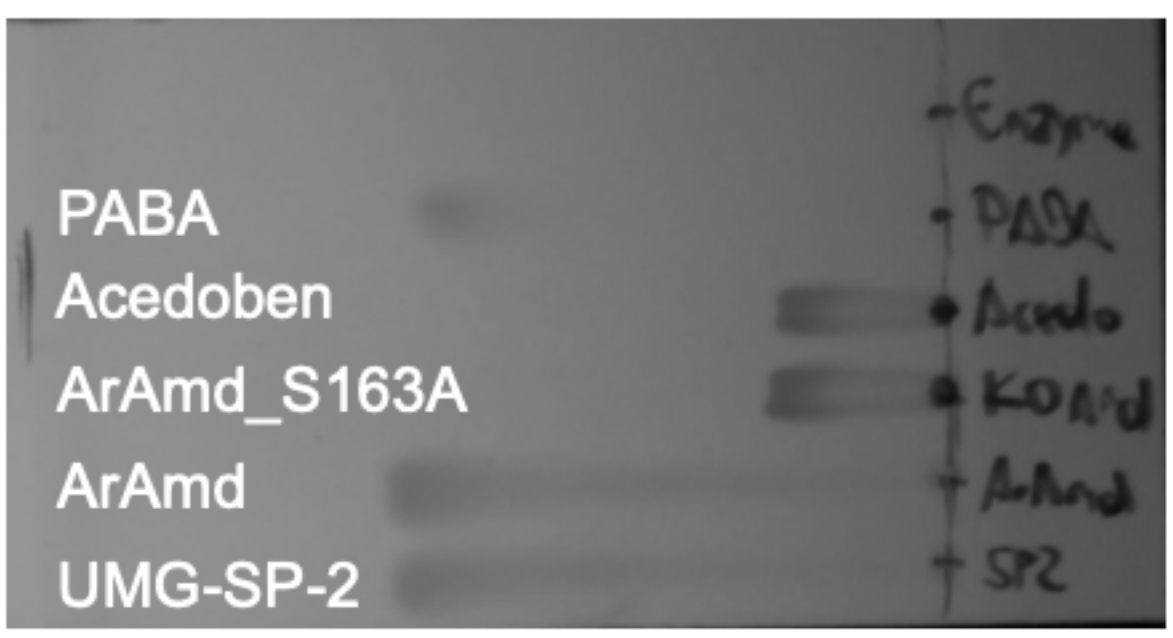

Fig. S3 Thin-layer chromatography of reaction products from the hydrolysis of acedoben using UMG-SP-2, ArAmd, and ArAmd_S163A together with the controls acedoben, PABA, and enzyme without substrate. While UMG-SP-2 and ArAmd fully converted acedoben to PABA, ArAmd_S163A did not.

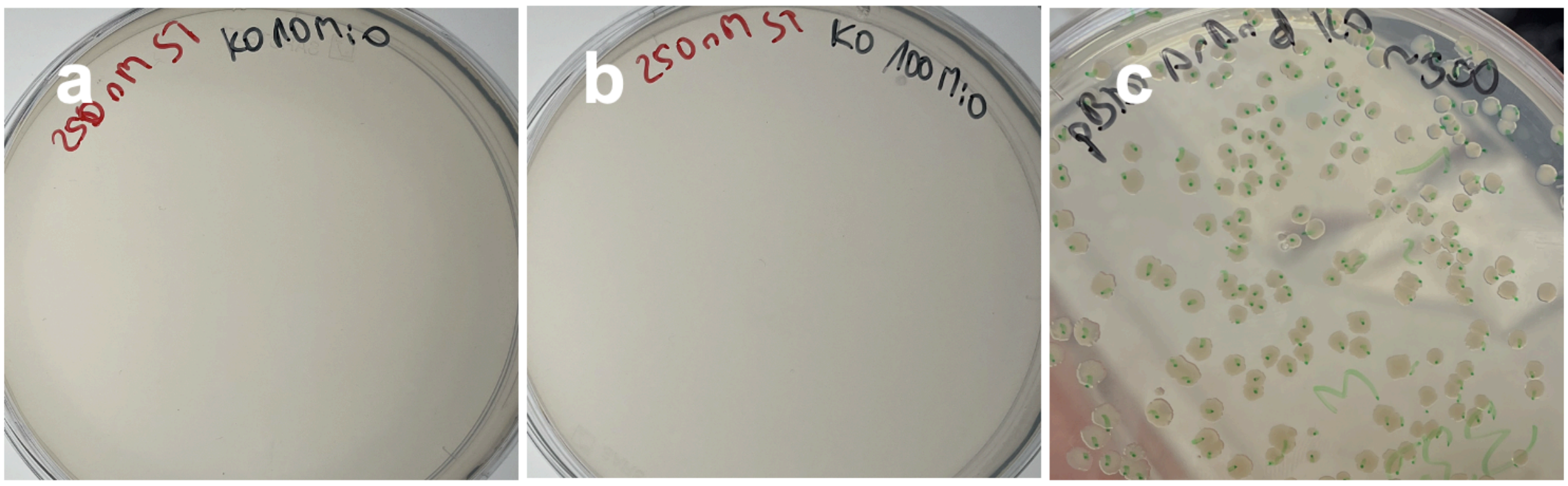

Fig. S4 Dilution series of Δ*pabA*_pBAD_*aramd*_S163A on screening plates containing 250 nM ST. Cell densities of up to ~6 million (**a**) and ~60 million (**b**) cells per plate were plated out without developing a cell lawn. The experiment was done in duplets and dilution series of the strain plated out on LB agar plates (example given in **c)** served as verification of cell density.

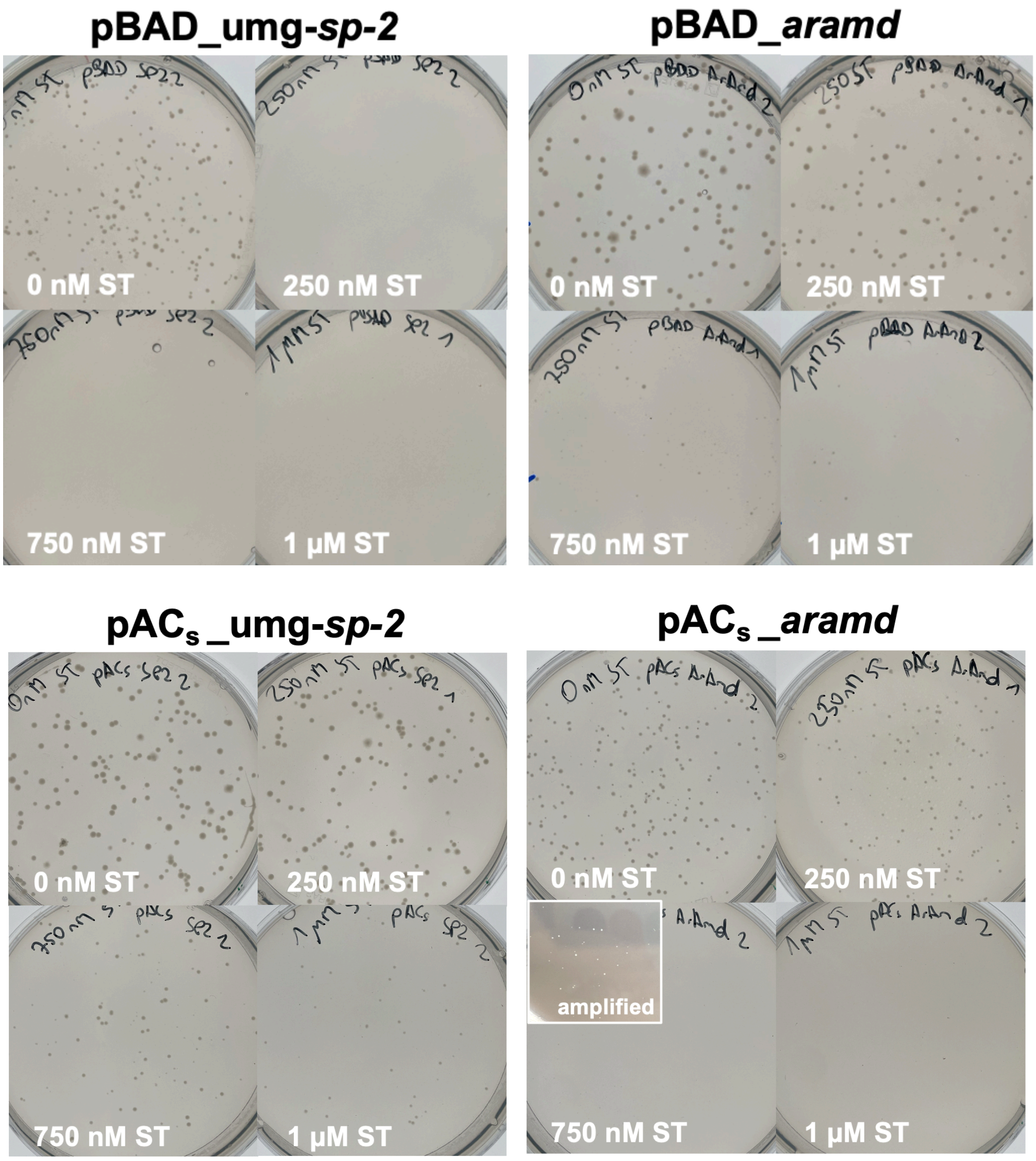

Fig. S5 Screening plates containing chloramphenicol (pAC$_s$) or ampicillin (pBAD) and different concentrations of ST, inoculated with Δ*pabA* carrying pBAD_*umg-sp-2* (upper left), pBAD_*aramd* (upper right), pAC$_s$_*umg-sp-2* (lower left), or pAC$_s$_*aramd* (lower right). Whereas the pAC$_s$-vector significantly increased the assay sensitivity for UMG-SP-2 compared to the pBAD vector, this was not the case for ArAmd, where the sensitivity even slightly decreased. The plates were inoculated with 100 µL of a 1:200,000-dilution of an overnight culture washed and resuspended to OD_{600} = 1.0 in screening buffer. The experiment was done in duplicates.

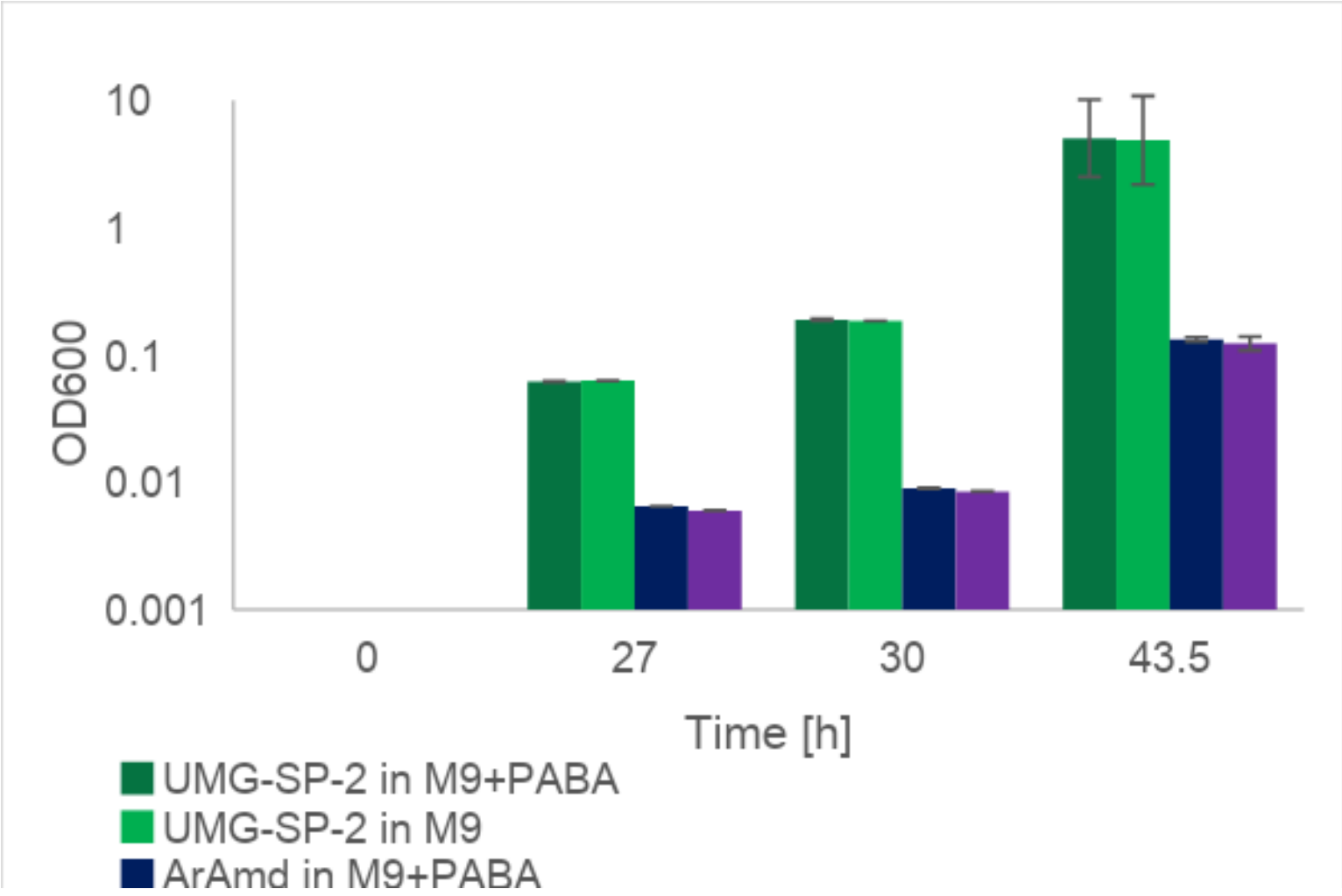

Fig. S6 Growth of Δ*pabA*_pAC$_s$_*aramd* and Δ*pabA*_pAC$_s$_umg-*sp-2* in screening medium (M9) and screening medium with an additional 5 µM PABA (M9+PABA). The strain expressing UMG-SP-2 showed significantly stronger growth compared to the strain expressing ArAmd, whereas the addition of PABA did not have any impact for both strains. The cultures had a volume of 20 mL and were inoculated 1:100,000 with log-phase pre-cultures normalized to an OD_{600} of 0.57. Strains were cultivated in duplicates.

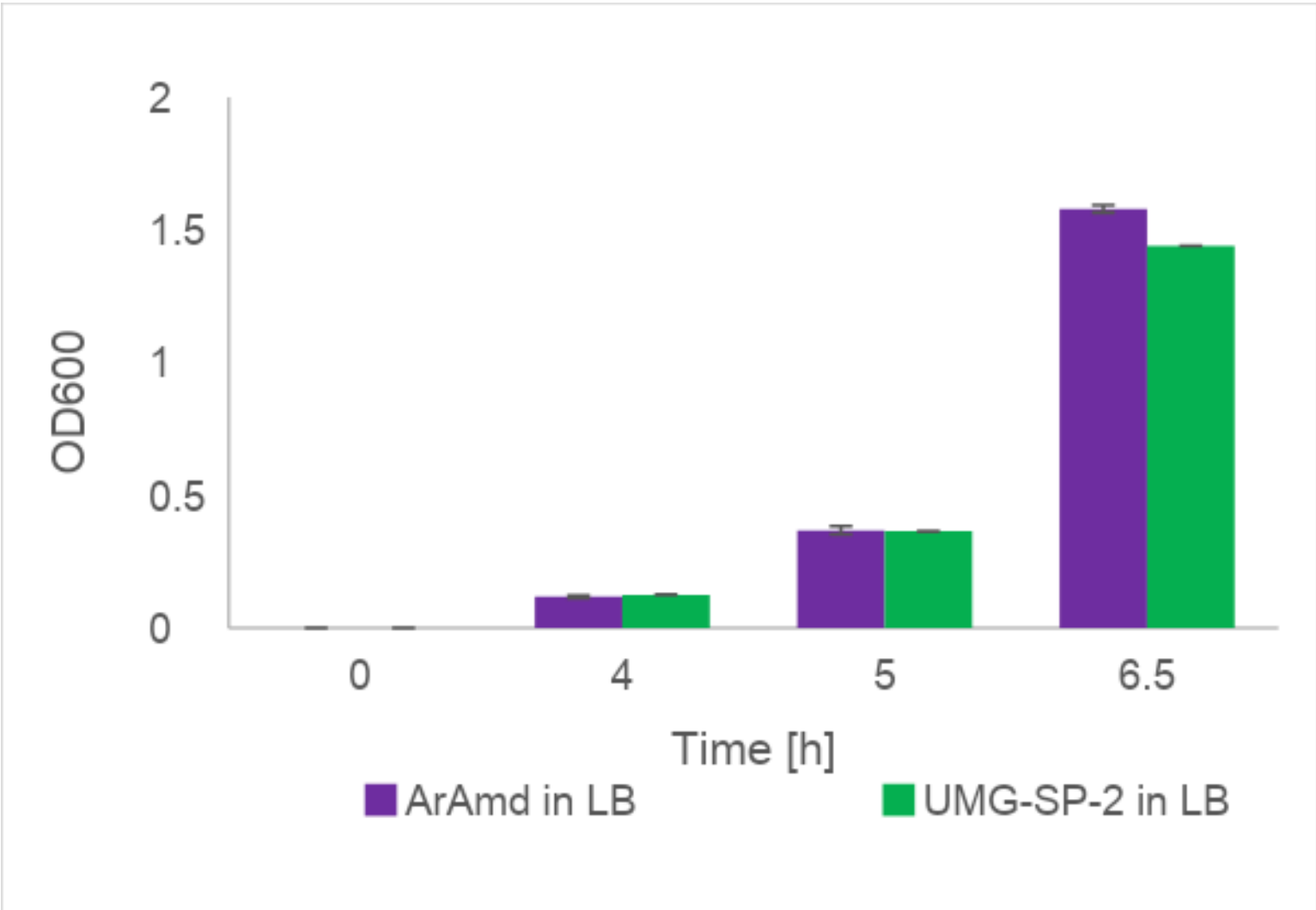

Fig. S7 Growth curve of Δ*pabA*_pAC$_s$_*aramd* and Δ*pabA*_pAC$_s$_*umg-sp-2* in LB medium. No difference in growth was visible between the strains within 6.5 h. The cultures had a volume of 30 mL and were inoculated 1:150 with log-phase pre-cultures normalized to an OD_{600} of 0.56. Strains were cultivated in duplicates.

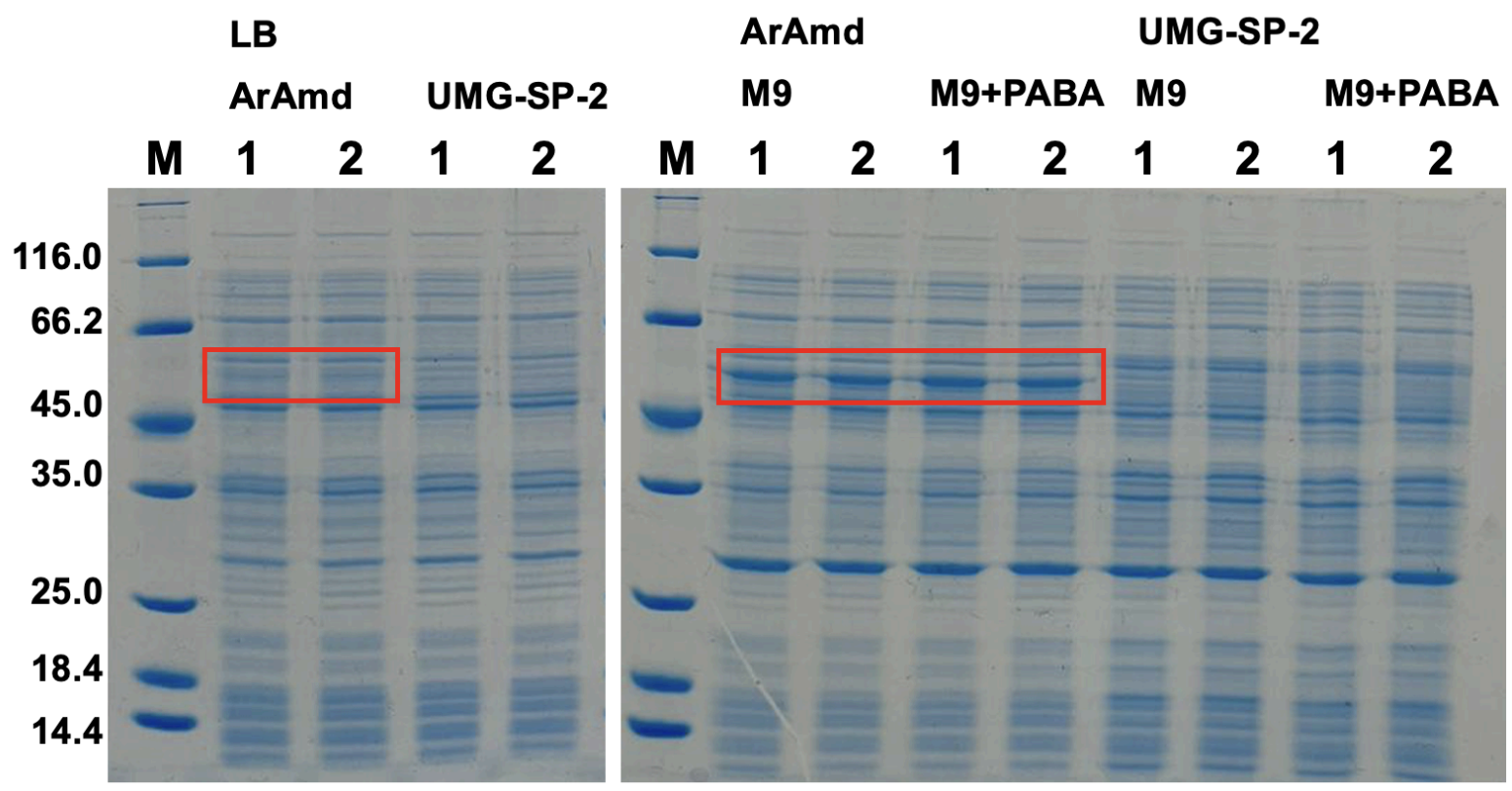

Fig. S8 SDS-PAGE analysis of cell cultures of Δ*pabA*_pAC$_s$_*aramd* and Δ*pabA*_pAC$_s$_*umg-sp-2* under different cultures conditions. From left to right: Frist gel: Marker (M), cultures in LB (1, 2). Faint bands of ArAmd were visible, no bands of UMG-SP-2 were visible after 6.5 h of incubation. Second gel: Marker (M), ArAmd cultures in screening medium (M9) and screening medium with 5 µM additional PABA (M9+PABA) in duplets (1, 2), UMG-SP-2 cultures in screening medium (M9) and screening medium with 5 µM additional PABA (M9+PABA) in duplets (1, 2). Strong bands of ArAmd and no bands of UMG-SP-2 were visible after 42 h of incubation. No difference was visible between screening medium and screening medium with additional PABA. All cultures were normalized to OD_{600} = 2.0.

DNA sequences and oligonucleotides

**pBAD_*umg-sp-2***	
pBAD_BBx_fw	AGCTTGGCTGTTTTGGCG
pBAD_BBx_rv	GGTTAATTCCTCCTGTTAGCC
pBAD_SP2_fw	gctaacaggaggaattaaccATGAGCGAGCTTTCCGCAATC
pBAD_SP2_rv	tccgccaaaacagccaagctTCAGTGGTGGTGGTGGTG
**pAC$_s$_*umg-sp-2***	
pAC_BB_fw	taatgcttaagtcgaacag
pAC_BB_rv	ggtatatctccttattaaagttaaac
pAC_SP2_fw	ctttaataaggagatataccatgagcgagctttccgcaatc
pAC_SP2_rv	tctgttcgacttaagcattatcagtggtggtggtggtg
**pAC$_s$_*aramd***	
pAC_BB_fw	taatgcttaagtcgaacag
pAC_BB_rv	ggtatatctccttattaaagttaaac
pAC_ArAmd_fw	ctttaataaggagatataccATGGGTAAAAGTCATAGTCCGG
pAC_ArAmd_rv	tctgttcgacttaagcattaTCAGTGGTGGTGGTGGTG
pBAD_*pabA*	
pbad_BB_HisTag_fw	CTCGAGCACCACCACCAC
pBAD_BB_*pabA*_rv	GGGTATGTATATCTCCTTCTTAAAGTTAAACAAAATTATTTC
*pabA*_insert_fw	AGAAGGAGATATACATACCCatgatcctgcttatagataactac
*pabA*_insert_rv	TGGTGGTGGTGGTGCTCGAGgcgatgcaggaaattagc

aramd

atgggtaaaagtcatagtccggtgcattggaaaagtgcagccgaaattgttgaactggttaaaagcaaacagattagcccgcgcgaagtggttgaaa
gtaccattgatctgattgaacagcgcgatccgggtctgaatgccgttgtgtataaagcatacgatgaagcccgtgaaaaagcagcagcactggaacg
tcgtattatgcagggtgaaccggtgggcatgctggcaggtgttccgaccctgatgaaagatctgttcgccgccaaaccgggttggccgagcaccct
gggtggcattcgtgccctgaaagatgcacgcggcgccgcaggcgtgtggtcaacatatccgctgaaaatgagcggtgaagatagtctgctgctgg
gccagaccaatagcccggtgtatggcttccgtggtaccaccgataataccttcttcggcccgacccgtaatccgttcaatctggacttcaatgcaggtg
gcagtagtggtggtgccgccgccctggtggcagatggtattgtgccggtggccggcggcaccgatggtggtggtagcattcgtattccggccgcct
ggaccaatacctatggcttccagccgagtattggtcgcgttccgttcaaaagtcgtccgaatgccttccatccgggcccgtatctgtatgaaggtccga
ttacccgtaccgtgcgcgatgccgccctggcaatgaatgttctgcatggcttcgatcgtcgtgatccggcaagcctgcgtgtgaaactggacttcacc
agcgcactggcacagggcgttcgtggcaaaaaaattggtctgaccctgaattatggtgtgttcccggtgcagcaggaaattcaggatctgattggtaa
agccgcacgcgtgttcaccgaactgggtgcacatgtggaattcgtggatctgggcattccgtatagtcagaaacagatgagtgatgcatggtgccgt
atgattgcaattccgaccgtggccagcatgcaggcactgcgcaaagaaggcattgatctgtatggtgaacatcgtgccgatattccggatgccctgat
gaaatggattgatgccgttgccgatattagcgttcagcagattagcgccgatcagctgctgcgtaccaccgtgttcgattgcatgaatggcgtgttcga
tcgcttcgatctgctgctggccccgaccctggcatgtatgccggttcgtaatgccaccgatggctgtaccgaaggcccgagtcagattaatggcgaa
gaaattgatccgctgattggttggtgcatgacctatctgaccaacttcagtggccatccgagtgcaagcgttccggccggcctgattgatggcctgcc
ggcaggcatgctgattattggcgatcgccaggccgatctggatgttattgcagcaagtgccgcattcgaacgtgcaagtccgtggagtcagtattatg
atattccggcaggtcgcccgctgctcgagcaccaccaccaccaccactga

*aramd*_S163A

atgggtaaaagtcatagtccggtgcattggaaaagtgcagccgaaattgttgaactggttaaaagcaaacagattagcccgcgcgaagtggttgaaa
gtaccattgatctgattgaacagcgcgatccgggtctgaatgccgttgtgtataaagcatacgatgaagcccgtgaaaaagcagcagcactggaacg
tcgtattatgcagggtgaaccggtgggcatgctggcaggtgttccgaccctgatgaaagatctgttcgccgccaaaccgggttggccgagcaccct
gggtggcattcgtgccctgaaagatgcacgcggcgccgcaggcgtgtggtcaacatatccgctgaaaatgagcggtgaagatagtctgctgctgg
gccagaccaatagcccggtgtatggcttccgtggtaccaccgataataccttcttcggcccgacccgtaatccgttcaatctggacttcaatgcaggtg
gcGCAagtggtggtgccgccgccctggtggcagatggtattgtgccggtggccggcggcaccgatggtggtggtagcattcgtattccggccg
cctggaccaatacctatggcttccagccgagtattggtcgcgttccgttcaaaagtcgtccgaatgccttccatccgggcccgtatctgtatgaaggtc
cgattacccgtaccgtgcgcgatgccgccctggcaatgaatgttctgcatggcttcgatcgtcgtgatccggcaagcctgcgtgtgaaactggacttc
accagcgcactggcacagggcgttcgtggcaaaaaaattggtctgaccctgaattatggtgtgttcccggtgcagcaggaaattcaggatctgattg
gtaaagccgcacgcgtgttcaccgaactgggtgcacatgtggaattcgtggatctgggcattccgtatagtcagaaacagatgagtgatgcatggtg
ccgtatgattgcaattccgaccgtggccagcatgcaggcactgcgcaaagaaggcattgatctgtatggtgaacatcgtgccgatattccggatgcc
ctgatgaaatggattgatgccgttgccgatattagcgttcagcagattagcgccgatcagctgctgcgtaccaccgtgttcgattgcatgaatggcgtg
ttcgatcgcttcgatctgctgctggccccgaccctggcatgtatgccggttcgtaatgccaccgatggctgtaccgaaggcccgagtcagattaatgg
cgaagaaattgatccgctgattggttggtgcatgacctatctgaccaacttcagtggccatccgagtgcaagcgttccggccggcctgattgatggcc
tgccggcaggcatgctgattattggcgatcgccaggccgatctggatgttattgcagcaagtgccgcattcgaacgtgcaagtccgtggagtcagtat
tatgatattccggcaggtcgcccgctgctcgagcaccaccaccaccaccactga

umg-sp-2

atgagcgagctttccgcaatcgaaaccgccgccgcgattgcgggcggtagcatgaccgcacttgaggcctgcgatgccgcgatcgcgcgcatcg
agcagcgcgacgggccgatcaatgccgtcgtcgttcgcgatttcgatcgcgcgcgcgacgcggcgaaggctgccgatgccgagatcgccgccg
ccgttcgcaagccgctgctcggcgtgccgatgacgatcaaggaatcgttcgacattgcggggctgccgacaagttggggcttcgccgaacatgcc
gatcacatcgccactgccgattcgctcgtcgtatcgcggctgaaggcagccggggccgtctttctcggcaagagcaacattcccgtcggacttgcc
gactggcagtcggtcaaccccaattacgggcgcaccaacaatccgcacgatcattcgcgcagcgccggcggctcatcgggcggtgcggcggcg
gcgctggcggcgggaatggtcccgctggaatatggctccgacattggcggttcgatccgcgtgccggcccatttctgcggcgtctggggtttgaag
acgaccttcgacgcagtcagtttggaaggtcattattttccgcgcaccgatagcgccaaggccgatctttcggtggtcggcccgatggcacggacgc
ccgccgacctcgctcttgcgctcgatatcacgagtaaggtgccacttccgcagtcgcgcatcgcaaatctttcggggctgcgcattctactgctgacg
gcacatcccgaaacggtcgccgatagcgcaacgatatcggcagtcgaaagagcagcggcagcctgcgaggcgagcggcgcgacggtcgcga
cgtcgagccccgacctgcccgacctgtccgcgctcgtcgccgattatacgcggatgttgctcgtcgtgcttgcgcgcggcctggcaccggaaggta
ccgagccggtcagtctgaatgcctggtatgcaatgcttgacgatcaggcgcggatgatgcgcgccttcgatcggctcttcgaaagtttcgacgcgatt
ttctgcccggttctcggcaccactgcctttgcccacagcgacgaacccgattgggccaagcggagcctgagcatcgacggcggcattgcgcccttc
gcggcgcaactcggctggatcagcatggcgacttatggcgggatgccggcgctgtctatgccgctcggcgctgatggcaacggcctgccgatcaa
ccttcagatcatcacgcgcaactggtccgatcatgatgcgatcaggatcggcgccttggtcgcggaagcgctcgaccgtctcgagcaccaccacca
ccaccactga

pabA

atgatcctgcttatagataactacgattcttttacctggaacctctaccagtacttttgtgaactgggggcggatgtgctggttaagcgcaacgatgcgtt
gacgctggcggatatcgacgcccttaaaccacaaaaaattgtcatctcacctggcccctgtacgccagatgaagccgggatctcccttgacgttattc
gccactatgccgggcgcttgccgattcttggcgtctgcctcggtcatcaggcaatggcgcaggcatttggcggtaaagttgtgcgcgccgcaaaggt
catgcacggcaaaacctcgccgattacacataacggtgagggcgtatttcgggggctggcaaatccacttaccgtgacacgctaccattcgctggtg
gtggaacctgactcattaccagcgtgctttgacgtgacggcctggagcgaaacccgagagattatggggattcgccatcgccagtgggatctggaa
ggtgtgcagttccatccagaaagtattcttagcgaacaaggacatcaactgctggctaatttcctgcatcgcctcgagcaccaccaccaccaccactg
a

pabB

atgaagacgttatctcccgctgtgattactttactctggcgtcaggacgccgctgaattttatttctcccgcttaagccacctgccgtgggcgatgcttta
cactccggctatgccgatcatccgtatagccgctttgatattgtggtcgccgagccgatttgcactttaaccactttcggtaaagaaaccgttgttagtga
aagcgaaaaacgcacaacgaccactgatgacccgctacaggtgctccagcaggtgctggatcgcgcagacattcgcccaacgcataacgaagat
ttgccatttcagggcggcgcactggggttgtttggctacgatctgggccgccgttttgagtcactgccagaaattgcggaacaagatatcgttctgccg
gatatggcagtgggtatctacgattgggcgctcattgtcgaccaccagcgtcatacagtttctttgctgagtcataatgatgtcaatgcccgtcgggcct
ggctggaaagccagcaattctcgccgcaggaagatttcacgctcacttccgactggcaatccaatatgacccgcgagcagtacggcgaaaaatttc
gccaggtacaggaatatctgcacagcggtgattgctatcaggtgaatctcgcccaacgttttcatgcgacctattctggcgatgaatggcaggcattcc
ttcagcttaatcaggccaaccgcgcgccatttagcgctttttacgtcttgaacagggtgcaattttaagcctttcgccagagcggtttattctttgtgataa

tagtgaaatccagacccgcccgattaaaggcacgctaccacgcctgcccgatcctcaggaagatagcaaacaagcagtaaaactggcgaactcag
cgaaagatcgtgccgaaaatctgatgattgtcgatttaatgcgtaatgatatcggtcgtgttgccgtagcaggttcggtaaaagtaccagagctgttcgt
ggtggaacccttccctgccgtgcatcatctggtcagcaccataacggcgcaactaccagaacagttacacgccagcgatctgctgcgcgcagctttt
cctggtggctcaataaccggggctccgaaagtacgggctatggaaattatcgacgaactggaaccgcagcgacgcaatgcctggtgcggcagcat
tggctatttgagcttttgcggcaacatggataccagtattactatccgcacgctgactgccattaacggacaaattttctgctctgcgggcggtggaatt
gtcgccgatagccaggaagaagcggaatatcaggaaacttttgataaagttaatcgtatcctgaagcaactggagaagctcgagcaccaccaccac
caccactga

Protein sequences of used enzymes

ArAmd

MGKSHSPVHWKSAAEIVELVKSKQISPREVVESTIDLIEQRDPGLNAVVYKAYDEAREKAAAL
ERRIMQGEPVGMLAGVPTLMKDLFAAKPGWPSTLGGIRALKDARGAAGVWSTYPLKMSGED
SLLLGQTNSPVYGFRGTTDNTFFGPTRNPFNLDFNAGGSSGGAAALVADGIVPVAGGTDGGGS
IRIPAAWTNTYGFQPSIGRVPFKSRPNAFHPGPYLYEGPITRTVRDAALAMNVLHGFDRRDPAS
LRVKLDFTSALAQGVRGKKIGLTLNYGVFPVQQEIQDLIGKAARVFTELGAHVEFVDLGIPYS
QKQMSDAWCRMIAIPTVASMQALRKEGIDLYGEHRADIPDALMKWIDAVADISVQQISADQLL
RTTVFDCMNGVFDRFDLLLAPTLACMPVRNATDGCTEGPSQINGEEIDPLIGWCMTYLTNFSG
HPSASVPAGLIDGLPAGMLIIGDRQADLDVIAASAAFERASPWSQYYDIPAGRPLLEHHHHHH*

ArAmd_S163A

MGKSHSPVHWKSAAEIVELVKSKQISPREVVESTIDLIEQRDPGLNAVVYKAYDEAREKAAAL
ERRIMQGEPVGMLAGVPTLMKDLFAAKPGWPSTLGGIRALKDARGAAGVWSTYPLKMSGED
SLLLGQTNSPVYGFRGTTDNTFFGPTRNPFNLDFNAGGASGGAAALVADGIVPVAGGTDGGG
SIRIPAAWTNTYGFQPSIGRVPFKSRPNAFHPGPYLYEGPITRTVRDAALAMNVLHGFDRRDPA
SLRVKLDFTSALAQGVRGKKIGLTLNYGVFPVQQEIQDLIGKAARVFTELGAHVEFVDLGIPYS
QKQMSDAWCRMIAIPTVASMQALRKEGIDLYGEHRADIPDALMKWIDAVADISVQQISADQLL
RTTVFDCMNGVFDRFDLLLAPTLACMPVRNATDGCTEGPSQINGEEIDPLIGWCMTYLTNFSG
HPSASVPAGLIDGLPAGMLIIGDRQADLDVIAASAAFERASPWSQYYDIPAGRPLLEHHHHHH*

UMG-SP-2

MSELSAIETAAAIAGGSMTALEACDAAIARIEQRDGPINAVVVRDFDRARDAAKAADAEIAAA
VRKPLLGVPMTIKESFDIAGLPTSWGFAEHADHIATADSLVVSRLKAAGAVFLGKSNIPVGLAD
WQSVNPNYGRTNNPHDHSRSAGGSSGGAAAALAAGMVPLEYGSDIGGSIRVPAHFCGVWGL
KTTFDAVSLEGHYFPRTDSAKADLSVVGPMARTPADLALALDITSKVPLPQSRIANLSGLRILL

LTAHPETVADSATISAVERAAAACEASGATVATSSPDLPDLSALVADYTRMLLVVLARGLAPEG
TEPVSLNAWYAMLDDQARMMRAFDRLFESFDAIFCPVLGTTAFAHSDEPDWAKRSLSIDGGIA
PFAAQLGWISMATYGGMPALSMPLGADGNGLPINLQIITRNWSDHDAIRIGALVAEALDRLEH
HHHHH*

PabA

MILLIDNYDSFTWNLYQYFCELGADVLVKRNDALTLADIDALKPQKIVISPGPCTPDEAGISLD
VIRHYAGRLPILGVCLGHQAMAQAFGGKVVRAAKVMHGKTSPITHNGEGVFRGLANPLTVT
RYHSLVVEPDSLPACFDVTAWSETREIMGIRHRQWDLEGVQFHPESILSEQGHQLLANFLHRLE
HHHHHH*

PabB

MKTLSPAVITLLWRQDAAEFYFSRLSHLPWAMLLHSGYADHPYSRFDIVVAEPICTLTTFGKET
VVSESEKRTTTTDDPLQVLQQVLDRADIRPTHNEDLPFQGGALGLFGYDLGRRFESLPEIAEQ
DIVLPDMAVGIYDWALIVDHQRHTVSLLSHNDVNARRAWLESQQFSPQEDFTLTSDWQSNMT
REQYGEKFRQVQEYLHSGDCYQVNLAQRFHATYSGDEWQAFLQLNQANRAPFSAFLRLEQG
AILSLSPERFILCDNSEIQTRPIKGTLPRLPDPQEDSKQAVKLANSAKDRAENLMIVDLMRNDIG
RVAVAGSVKVPELFVVEPFPAVHHLVSTITAQLPEQLHASDLLRAAFPGGSITGAPKVRAMEIID
ELEPQRRNAWCGSIGYLSFCGNMDTSITIRTLTAINGQIFCSAGGGIVADSQEEAEYQETFDKVN
RILKQLEKLEHHHHHH*

Reference

Lee Y, Umeano A, Balskus EP (2013) Rescuing auxotrophic microorganisms with nonenzymatic chemistry. *Angew. Chem. Int. Ed*. **52**:11800–11803. https://doi.org/10.1002/anie.201307033
